# Conditional Acceptance and the Optimism–Knowledge Gap: A Scoping Review of Attitudes and Perceptions of Artificial Intelligence in Healthcare in Italy

**DOI:** 10.3390/medsci14020276

**Published:** 2026-05-28

**Authors:** Christian J. Wiedermann, Giuliano Piccoliori, Doris Hager von Strobele Prainsack, Dietmar Ausserhofer

**Affiliations:** 1 Institute of General Practice and Public Health, Claudiana—College of Health Professions, 39012 Bolzano, Italy; 2 Claudiana Research, Claudiana—College of Health Professions, 39012 Bolzano, Italy

**Keywords:** scoping review, artificial intelligence, healthcare, Italy, attitudes, acceptance, perceptions, healthcare professionals, PRISMA-ScR, South Tyrol

## Abstract

**Background/Objectives**: Artificial intelligence (AI) is integrated into diagnostic, therapeutic, administrative, and communicative healthcare domains in Italy under regulations requiring human oversight. Empirical evidence on AI attitudes, acceptance, and perceptions in Italian healthcare is rapidly accumulating but not systematically mapped. This scoping review aimed to (i) map empirical evidence on AI attitudes, acceptance, and perceptions in Italy by population and domain; (ii) identify measurement instruments used in studies and their origins; and (iii) characterize determinants, themes, and methodological gaps in the Italian evidence base. **Methods:** This review used Joanna Briggs Institute methodology, reported via PRISMA-ScR (protocol Open Science Framework doi: 10.17605/OSF.IO/TZRVF). PubMed and Embase were searched on 27 April 2026 from January 2018 in English, Italian, or German, combining controlled vocabulary and free-text terms across AI, attitudes-acceptance, and healthcare delivery, with an Italian-context qualifier; a complementary AI-assisted semantic search (Consensus Pro) was conducted to validate retrieval completeness. Eligibility criteria used the Population–Concept–Context mnemonic. **Results:** Of 1510 unique records screened, 35 empirical studies were retained, comprising 7 studies of Italian patients and the general population, 22 studies of healthcare professionals, 3 psychometric validation studies of AI-acceptance instruments, 1 mixed-population study, and 2 international comparator studies with substantial Italian sub-samples. Acceptance was consistently positive but conditional on physician oversight, training, and regulatory clarity. A recurrent optimism–knowledge gap and an absence of probabilistic, population-representative evidence were identified as principal gaps. **Conclusions:** Italian evidence on AI attitudes is expanding but methodologically narrow. Three Italian-validated acceptance instruments are now available. Population-representative, multilingual, and longitudinal evidence is required.

## 1. Introduction

AI has transitioned from research to clinical practice in diagnostic, therapeutic, administrative, and communicative healthcare. Large language models, computer-vision algorithms for medical imaging, AI-enabled symptom checkers, clinical decision-support systems, and conversational agents are used in hospitals and patient services in high-income countries. This deployment has accelerated since 2018, with generative-AI tools in 2022 affecting clinical workflows, roles, patient information, and doctor–patient relationships beyond technical performance [1].

Successful AI integration requires more than algorithmic accuracy. Technology adoption research shows uptake is influenced by users’ beliefs, attitudes, and the organizational context. The Technology Acceptance Model [2], Unified Theory of Acceptance and Use of Technology [3], and AI-specific frameworks like the Artificially Intelligent Device Use Acceptance [4] view acceptance as a result of perceived usefulness, ease of use, social influence, and in AI, anthropomorphism, hedonic motivation, and emotion. Reviews emphasize organizational complexity, professional identity, and institutional trust affect acceptance, not fully captured by original models [5,6]. Acceptance is a multidimensional construct including trust, perceived risks and benefits, willingness to use, concerns about autonomy, accountability, and AI literacy.

Constructs and their distribution differ across stakeholders. Patients and the public evaluate AI through trust, reliability, data protection, and human contact in care; acceptance requires physician oversight and transparency [7]. Physicians and healthcare professionals assess AI’s clinical utility, autonomy, liability, and doctor–patient relationship; surveys show cautious optimism with concerns about regulation, training, and de-professionalization [7,8,9]. Nurses, allied health professionals, and trainees worry about dehumanization, workflow disruption, and skill erosion [10]. The European regulatory landscape adds complexity. The General Data Protection Regulation (GDPR) influences AI use, and the EU’s Artificial Intelligence Act classifies medical AI as high-risk, requiring transparency, oversight, robustness, and monitoring [11,12]. Implementation needs sector-specific clarification, and interaction between the AI Act, national health laws, and professional norms is debated [13].

Italy holds a distinctive position in Europe along three axes that, in combination, are not present in comparable healthcare systems. Despite having one of the EU’s largest healthcare systems, its AI investment is modest within a context where AI adoption is uneven and focused on well-funded organizations [14,15]. Deployment in Italy is mainly in academic centers and regional initiatives, often funded by the National Recovery and Resilience Plan (Piano Nazionale di Ripresa e Resilienza–PNRR) and in collaboration with research institutes or tech providers [15]. Italy’s data protection regime is stringent: the “Garante per la protezione dei dati personali”, Italy’s national Data Protection Authority, classifies healthcare AI as high-risk, requiring clinician oversight of AI suggestions [16]. These provisions translate physician oversight from a normative preference into a regulatory precondition for AI deployment in the National Health Service, a configuration not present in most European comparator jurisdictions. Law 132/2025 formalizes these requirements within the National Health Service [17]. Italy is additionally characterized by pronounced regional and linguistic heterogeneity, including German- and Ladin-speaking communities in the Autonomous Province of Bolzano-South Tyrol and further language groups in Friuli-Venezia Giulia and Valle d’Aosta—a configuration that shapes both AI-mediated communication and the design of evidence on its acceptance. The PNRR and the National Agency for Regional Health Services (Agenzia Nazionale per i Servizi Sanitari Regionali–AGENAS) fund AI infrastructure for primary care [18], and the 2025 Ministry of Health guidelines call for AI integration into national systems [17]. This regulatory environment makes clinician competence and population literacy preconditions for compliance rather than aspirational targets.

Research on AI attitudes in Italian healthcare has grown, covering patients, physicians, residents, allied health professionals, and nurses. Italian versions of acceptance instruments are emerging. However, systematic mapping is lacking. The focus on stakeholder groups, clinical applications, instruments, acceptance determinants, and gaps—like population-level evidence, multilingual studies, and underrepresented groups—remains unclear.

This scoping review addresses this gap with three objectives: (i) map empirical evidence on AI attitudes, acceptance, and perceptions in Italy by population and clinical domain; (ii) identify measurement instruments in Italian studies and their psychometric origins; and (iii) characterize determinants and themes in Italian evidence, highlighting methodological and substantive gaps for further research.

## 2. Materials and Methods

### 2.1. Study Design and Reporting Framework

This scoping review mapped evidence on attitudes, acceptance, and perceptions of AI in Italian healthcare among populations and professionals, identifying gaps for further study. This approach was chosen for its effectiveness in examining a diverse evidence base, clarifying concepts, and guiding future research [19]. This review followed Arksey and O’Malley’s framework [20], refined by Levac et al. [21], and Joana Briggs Institute (JBI) Manual guidance [22]. Reporting follows PRISMA-ScR guidelines [23]; the checklist is in Supplementary Table S1.

The protocol of this scoping review was registered retrospectively with the Open Science Framework on 5 May 2026 (https://doi.org/10.17605/OSF.IO/TZRVF), after execution of the systematic search on 27 April 2026.

### 2.2. Review Questions

Review questions used the Population–Concept–Context (PCC) mnemonic by JBI for scoping reviews [4]. The main question was the following: what is known about AI in healthcare attitudes, acceptance, and perceptions in Italy, and what gaps justify further research in a multilingual context? The Population included the general adult population, patients, and healthcare professionals (physicians, nurses, allied health professionals, and students). The Concept included attitudes, acceptance, perceptions, trust, concerns, willingness, readiness, and literacy regarding AI in healthcare delivery, including diagnostic, therapeutic, administrative, and communicative applications. The Context involved studies in Italy or with Italian populations, alongside key international comparator studies for the South Tyrolean survey. For this review, the three core constructs were defined using the mentioned technology-acceptance frameworks. “Attitudes” refer to evaluative orientations towards AI in healthcare, covering positive and negative assessments along affective and cognitive dimensions. “Acceptance’ indicates willingness, intention, or behavioral endorsement of AI in healthcare, possibly contingent on safeguards like physician oversight. ‘Perceptions” involve subjective appraisals of AI attributes such as usefulness, ease of use, reliability, trustworthiness, and risk, distinct from overall acceptance. Studies qualified if they reported primary data on these constructs or related ones like trust, concerns, willingness, readiness, or AI literacy.

### 2.3. Eligibility Criteria

Eligibility criteria were specified a priori and applied uniformly across sources. Inclusion criteria included (i) empirical studies of any design reporting primary data on attitudes, acceptance, perceptions, trust, concerns, willingness, readiness, or literacy regarding AI in healthcare, in adult populations (individuals aged 18 years or older) or in mixed populations where adult sub-samples were separately analyzable; (ii) validation studies of measurement instruments for these constructs; (iii) studies conducted in Italy (populations resident in Italy during data collection, regardless of nationality or ethnicity), or with a substantial Italian sub-sample separately analyzable within a multinational design; studies of Italian-origin populations abroad were not eligible; (iv) publications from January 2018 to April 2026, coinciding with the diffusion of clinically deployable AI applications and large language models; and (v) articles in English, Italian, or German. Eligible study designs included quantitative observational studies (cross-sectional surveys, cohort studies, registry analyses), qualitative designs (semi-structured interviews, focus groups, reflexive thematic analyses), mixed-methods designs, randomized and non-randomized experimental survey studies, feasibility and pilot studies with empirical user evaluation, and psychometric validation studies of relevant instruments. Consistent with JBI guidance for scoping reviews, no methodological filter excluding particular designs was applied [19,22]. Eligible AI applications included systems based on machine learning, deep learning, large language models, generative AI, computer-vision algorithms for medical imaging, AI-enabled clinical decision-support systems, AI-based symptom checkers, chatbots, and AI components of assistive robotics, applied to diagnostic, therapeutic, administrative, or communicative functions in healthcare. No restriction was placed on the platform or mode; computer-based, web-based, mobile-based, and embedded-device applications were equally eligible. Rule-based decision-support systems without a learning component, and platforms where AI was peripheral or invoked only programmatically, were excluded at the full-text stage.

Exclusion criteria included (i) studies on AI development, validation, or technical performance without measuring attitudes or acceptance; (ii) editorials, commentaries, and opinion pieces without empirical or systematic components; (iii) studies on AI applications outside healthcare; (iv) conference abstracts without full-length publications of the same study, as resolved in Section 2.6; and (v) systematic, scoping, narrative reviews, and other secondary syntheses not providing primary empirical data on AI in healthcare attitudes or perceptions.

### 2.4. Information Sources

Three information sources were combined to balance bibliographic completeness with semantic coverage. PubMed served as the primary biomedical database; Embase was searched for extended European and clinically applied coverage, including records not indexed in MEDLINE; and Consensus Pro was queried as a complementary AI-assisted semantic search engine indexing approximately 200 million peer-reviewed papers across disciplines. The combined use of PubMed and Embase represents the conventional standard for evidence syntheses in the health sciences, while the addition of Consensus Pro served to broaden thematic retrieval beyond the indexing logic of formal databases and to validate the topical completeness of the database results. Searches were executed 27 April 2026. PubMed records were exported in RIS format and imported into Zotero 9.0.4 for downstream management. Embase records, where direct RIS export was not available through the institutional access channel used, were captured through structured Zotero reports preserving title, abstract, authorship, source, and indexing information for all retrieved records. All records were managed in Zotero, where deduplication, screening, and full-text retrieval were carried out.

### 2.5. Search Strategy

The search strategy was developed iteratively. An initial limited search of PubMed and Embase identified candidate index and free-text terms in titles, abstracts, and indexing fields of relevant articles. These terms were combined into the full search strategies reported below. In line with JBI guidance for scoping reviews, no methodological filter was applied to retain heterogeneity of study designs [19,22].

#### 2.5.1. PubMed

The PubMed search combined three blocks (artificial intelligence; attitudes, acceptance, and perceptions; healthcare delivery), limited to Italian context records and publications from 2018 onward. MeSH terms were combined with title/abstract terms, and an Affiliation qualifier was included to capture studies by Italian-based researchers regardless of geographic indexing. The full search string is reported verbatim:

((((“artificial intelligence”[MeSH Terms] OR “artificial intelligence”[tiab] OR “machine learning”[tiab] OR “deep learning”[tiab] OR “large language model*”[tiab] OR “generative AI”[tiab] OR “ChatGPT”[tiab]) AND (“attitude”[MeSH Terms] OR “attitudes”[tiab] OR “acceptance”[tiab] OR “perception*”[tiab] OR “trust”[tiab] OR “concerns”[tiab] OR “willingness”[tiab] OR “readiness”[tiab] OR “literacy”[tiab]) AND (“delivery of health care”[MeSH Terms] OR “health care”[tiab] OR “healthcare”[tiab] OR “medicine”[tiab] OR “clinical”[tiab] OR“primary care”[tiab] OR “hospital”[tiab] OR “nursing”[tiab])) AND (“Italy”[MeSH Terms] OR “Italy”[Affiliation] OR “Italian”[tiab] OR “Italy”[tiab] OR “Italia”[tiab])) AND (“2018”[Date-Publication]:“3000”[Date-Publication]))(1)

On 27 April 2026, the PubMed search yielded 729 records, all of which were exported in nbib format and imported into Zotero for downstream screening.

#### 2.5.2. Embase

Embase’s search mirrored PubMed’s structure, using Emtree-controlled vocabulary with title/abstract/keyword and author-affiliation terms. It retrieved records not in MEDLINE, focusing on clinical and applied research from Italian institutions. The full search expression is reported verbatim:

(‘artificial intelligence’/exp OR ‘machine learning’/exp) AND (‘attitude’/exp OR ‘patient attitude’/exp OR acceptance:ti,ab,kw OR perception*:ti,ab,kw) AND (‘health care’/exp OR ‘medicine’/exp) AND (‘italy’/exp OR italy:ti,ab,kw,af OR italian:ti,ab,kw,af) AND [2018–2026]/py(2)

The Embase search yielded 1011 records on 27 April 2026.

#### 2.5.3. Consensus Pro

To complement formal-database searches and validate retrieval completeness, an AI-assisted semantic search was conducted in Consensus Pro Analysis 9 February 2024 (San Francisco, CA, USA). Retrieval was limited to the platform’s “Medical” domain, focusing on the peer-reviewed health and biomedical literature. Instead of a single Boolean query, the topic was divided into nine research questions, each addressing a specific construct facet; for each, the top fifty relevant references were exported as separate reports.

The questions covered the following: digital and AI literacy as attitude determinants; perceptions of general practitioners and primary care physicians; attitudes, acceptance, and concerns of patients and the general population; key concerns of physicians and nurses regarding AI in clinical decision-making; evidence from Italy and other European countries on healthcare professionals’ attitudes toward AI, including studies on the EU AI Act and regional implementation; acceptance and use of AI in healthcare across different groups; current acceptance, attitudes, and perceptions toward AI among physicians and associated factors; attitudes toward AI in various specialties and settings; and clinical application areas (administrative, diagnostic, therapeutic, communicative) where AI is most accepted.

Consensus Pro outputs served as a complementary mapping aid rather than a primary evidence stream. Records identified through Consensus Pro and not retrieved by PubMed or Embase were checked against formal databases before inclusion.

### 2.6. Selection of Sources of Evidence

**Figure 1:**
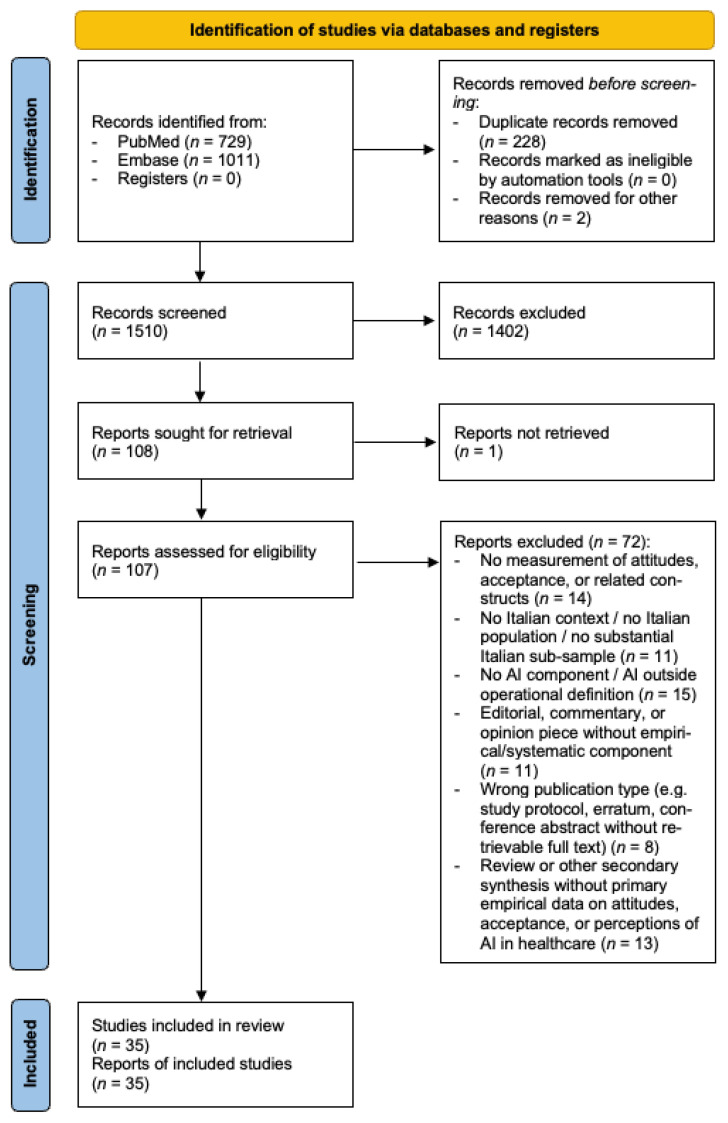
PRISMA 2020 flow diagram of the study selection process for the scoping review on attitudes, acceptance, and perceptions of artificial intelligence in healthcare in Italy. The diagram reports the identification, screening, and inclusion of records retrieved through formal database searching of PubMed and Embase (January 2018–April 2026). A complementary AI-assisted semantic search (Consensus Pro) served as a retrieval-completeness check rather than as a primary evidence stream and is not depicted in the flow diagram; records identified through Consensus Pro and not retrieved by PubMed or Embase were checked against the formal databases before inclusion. Records were excluded at the title and abstract stage for not measuring attitudes, acceptance, or related constructs (often technical AI studies), lacking an Italian context, population, or affiliation, addressing non-healthcare AI applications, or being editorials, commentaries, or secondary syntheses. Adapted from Page et al. [24]; reproduced and adapted under the Creative Commons Attribution 4.0 International License (CC BY 4.0).

Records from PubMed and Embase were imported into Zotero for deduplication via automatic detection and manual verification. Two records were excluded as retracted. The screening process is summarized in Figure 1. Title and abstract screening was conducted by the lead reviewer using the criteria in Section 2.3, with a conservative inclusion threshold. A random 20% of records were re-screened by a second reviewer to estimate inter-rater reliability, with high agreement; discrepancies were resolved by consensus, retaining the lead reviewer’s decisions where confirmed.

Records progressing beyond screening included full-length articles and conference abstracts. Per JBI guidance [22], where a conference abstract corresponded to a later full-length publication of the same study, the full-length publication was retrieved through supplementary Google Scholar searches and substituted for the abstract. Conference abstracts without an identifiable corresponding full publication were assessed for eligibility on their own merits and excluded where they did not provide primary empirical data on the attitudes-and-acceptance constructs in an Italian context. Detailed eligibility decisions and exclusion reasons for all records advanced to full-text screening are reported in Supplementary Table S2 [25,26,27,28,29,30,31,32,33,34,35,36,37,38,39,40,41,42,43,44,45,46,47,48,49,50,51,52,53,54,55,56,57,58,59].

Reasons for exclusion [60,61,62,63,64,65,66,67,68,69,70,71,72,73,74,75,76,77,78,79,80,81,82,83,84,85,86,87,88,89,90,91,92,93,94,95,96,97,98,99,100,101,102,103,104,105,106,107,108,109,110,111,112,113,114,115,116,117,118,119,120,121,122,123,124,125,126,127,128,129,130,131,132,133] at full-text assessment included no measurement of attitudes, acceptance, or related constructs; no Italian context, population, or substantial sub-sample; no AI component or applications outside the operational definition; editorials, commentaries, or opinion pieces lacking empirical or systematic components; incorrect publication types, such as study protocols and errata; and reviews and other secondary syntheses, which were considered the background literature but not primary evidence for the mapping objectives of this scoping review.

### 2.7. Data Charting Process

A draft data-charting form was developed iteratively per JBI guidance and pilot-tested on ten records before full extraction [22]. It captured bibliographic information (authors, year, journal, country); study design and setting; population characteristics (sample size, professional group, recruitment); measurement instruments and constructs (e.g., the Artificially Intelligent Device Use Acceptance scale (AIDUA), the General Attitudes towards Artificial Intelligence Scale (GAAIS), the Italian Knowledge, Attitudes, Practices and Clinical Application of Medical AI Questionnaire (I-KAPCAM-AI-Q) instrument, and ad hoc instruments); main findings on acceptance, attitudes, and perceptions; reported determinants and correlates; and methodological limitations by authors. Charted data produced a descriptive synthesis by population type (general population and patients; healthcare professionals; healthcare students) and application domain (diagnostic, therapeutic, administrative, communicative).

### 2.8. Synthesis of Results

In line with scoping reviews, no formal critical appraisal or risk-of-bias assessment was performed; the goal was to map evidence, not estimate effect sizes [19,22]. Synthesis was descriptive, with tabular summaries of study characteristics and a narrative of themes, instruments, and gaps. Frequencies of study designs, populations, domains, and instruments were tabulated. Recurrent thematic labels used to organize the descriptive synthesis (“conditional acceptance”, “optimism–knowledge gap”, “trust-mediated acceptance”) were derived from the included studies through a structured coding procedure. A label was retained only where the underlying construct was reported in three or more independent primary studies, where the numerical or qualitative evidence across those studies converged in direction, and where the construct was named explicitly by at least one primary study within the corpus. The label “optimism–knowledge gap” was adopted from the wording of Perrella et al. [25]; “conditional acceptance” and “trust-mediated acceptance” were coded inductively across the corpus. Operational definitions are provided at first use in the Results. These labels function as organizing categories for the descriptive synthesis and are not advanced as established theoretical constructs. Themes on trust, perceived risks, benefits, and educational needs were grouped narratively across main population strata.

## 3. Results

### 3.1. Selection of Sources of Evidence and Characteristics of Included Studies

The selection process is summarized in Figure 1. The combined PubMed and Embase searches retrieved 1740 records; after deduplication and removal of two retracted publications, 1510 unique records were screened on title and abstract, 107 were assessed in full text, and 35 empirical studies were retained for synthesis. Full-text decisions and exclusion reasons for all 107 records are reported in Supplementary Table S2 [25,26,27,28,29,30,31,32,33,34,35,36,37,38,39,40,41,42,43,44,45,46,47,48,49,50,51,52,53,54,55,56,57,58,59,60,61,62,63,64,65,66,67,68,69,70,71,72,73,74,75,76,77,78,79,80,81,82,83,84,85,86,87,88,89,90,91,92,93,94,95,96,97,98,99,100,101,102,103,104,105,106,107,108,109,110,111,112,113,114,115,116,117,118,119,120,121,122,123,124,125,126,127,128,129,130,131,132,133].

The 35 included studies are summarized in Table 1 and grouped into five strata. Seven studies addressed Italian general-population and patient perspectives (Class A1) [26,27,28,29,30,31,32]; 22 studies addressed Italian healthcare professionals (Class A2) [25,33,34,35,36,37,38,39,40,41,42,43,44,45,46,47,48,49,50,51,52,53]; three studies were Italian psychometric validation studies of acceptance instruments (Class A3) [54,55,56]; one study combined Italian and Dutch populations within a single mixed-design needs assessment (Class B) [57]; and two studies were international comparator studies with a substantial Italian sub-sample within multi-country European cancer-care designs (Class C) [58,59].

The temporal distribution of the corpus is heavily skewed towards the most recent years. Four studies were published in 2021 [35,39,40,58] and four in 2023 [29,30,52,59]. A further four studies appeared in 2024 [27,36,37,60], and the remainder (*n* = 23, equivalent to 65.7% of the corpus) were published during 2025 (*n* = 10) [28,42,43,44,45,47,50,51,56,57] and the first four months of 2026 (*n* = 13) [26,31,32,33,34,38,41,45,47,48,52,53,54]. The temporal concentration of the evidence base coincides with the diffusion of generative-AI applications into clinical practice and with the regulatory clarification of the European Artificial Intelligence Act [14], and it implies that almost two-thirds of the empirical evidence on AI acceptance in Italian healthcare has accumulated within the eighteen months preceding the closing date of the search.

Geographically, twenty studies were conducted at the national level, frequently through professional-society or national institution networks [26,28,33,34,35,42,44,46,49]. Four studies addressed regional populations or institutional clusters (Lombardy, Tuscany, Northern Italy, and the University of Padova) [37,38,49,54]. Five studies were geographically delimited to single cities, with three centered on academic radiology and oncology centers in Milan [30,31,32], one on the Istituto di Ricovero e Cura a Carattere Scientifico (IRCCS) Santa Lucia in Rome [27], and one on the IRCCS Istituto Romagnolo per lo Studio dei Tumori (IRST) Dino Amadori in Meldola [26]. One study was situated in the Autonomous Province of Bolzano in South Tyrol [29]. One study assembled a multidisciplinary sample of clinicians from Northern, Central, and Southern Italy at the Annual Thinking Lab on Fibromyalgia Syndrome (ATLAS) 2024 congress in Gubbio [36]. One study was a Campania-led national survey [45]. The three remaining studies [57,58,59] included Italian sub-samples within multinational European designs.

Cross-sectional surveys were the predominant design (*n* = 23 studies). Four studies adopted qualitative interview or reflexive thematic-analysis designs [41,49,58,60]. Two studies used mixed-methods designs [29,58], three were psychometric validation studies of acceptance instruments [54,55,56], and the remainder comprised one feasibility study with clinician evaluation [40], one randomized experimental survey [32], and one cross-sectional pre- and post-workshop survey [36].

Sample sizes ranged from 14 expert qualitative researchers [41] and 21 clinicians evaluating a conversational mental-health agent [40] to nationally representative population panels of 1200 [28] and 1540 pediatricians [51] and to a single-institution cohort of 1454 volunteers and neurological patients [27]; the cumulative empirical base across all 35 studies amounts to approximately 12,000 individual respondents from patient, professional, student, and trainee populations.

AI application domains ranged across diagnostic imaging and radiology (*n* = 7 studies) [30,31,32,34,37,39,46]; chatbots, large language models, and clinical decision-support tools deployed across primary care, gastroenterology, microbiology, neurology, nursing, rheumatology, mental-health care, physiotherapy, and digital pathology (*n* = 12 studies) [29,33,36,38,40,42,43,44,45,47,50,54]; patient-facing applications in cancer care, screening, and biobanking (*n* = 5 studies) [26,27,28,58,59]; psychometric instruments measuring AI acceptance constructs (*n* = 3 studies) [54,55,56]; and cross-cutting studies addressing general AI literacy, assistive robotics, AI-enhanced care, and qualitative inquiry into AI itself (*n* = 8 studies) [35,41,45,48,49,51,52,57].

### 3.2. Italian General-Population and Patient Perspectives (Class A1)

Seven studies focused on Italian patients’ and the general population’s views on AI in healthcare [26,27,28,29,30,31,32]. The corpus includes a national panel of 1200 respondents [28], a cohort of 1454 volunteers and neurological patients [27], a multicenter evaluation with 116 primary care patients [29], a survey of 117 oncology patients [26], and three surveys with 800 [30], 240 [31], and 600 [32] respondents at the European Institute of Oncology. Six studies are quantitative cross-sectional surveys; one is a mixed-methods feasibility evaluation of an AI symptom checker [29], and one survey involved a randomized design with mammography scenarios [32]. Except for La Regina 2025, which includes AI within a broader digital-health framework [28], all other studies focus on patient-level AI attitudes.

AI awareness and self-reported knowledge among Italian patients and the general population vary by age and education. Cavallucci 2026 found 70.1% of oncology patients had moderate AI awareness, with 85.5% aware of medical AI, higher among younger, educated respondents [26]. In the Pesapane 2023 mammography cohort, 51% of women reported accurate AI knowledge, with higher education and non-Italian background associated with more positive AI views [30]. Conversely, La Regina 2025 noted limited public knowledge of patient safety, clinical trials, and telemedicine among 1200 Italians, with no significant difference between 400 with severe chronic conditions and 800 without; better culture was linked to age above 44 and high education [28]. These results show AI literacy stratification by age, education, and nationality across seven A1 studies, with patterns at national [28], regional [26], and single-institution [30] levels.

In the descriptive synthesis that follows, “conditional acceptance” denotes positive endorsement of AI in healthcare contingent on the explicit retention of physician oversight or comparable institutional safeguards; “trust-mediated acceptance” denotes variation in acceptance that tracks the modality of AI disclosure or the perceived transparency of AI outputs rather than AI involvement per se. Across the Class A1 studies, acceptance of AI in healthcare was consistently positive but was reported as conditional on physician oversight. In the Pesapane 2026 survey, 96% of 240 respondents supported AI for assisting radiologists, 92% for diagnostics and treatments, and 90.4% had a positive view of AI in healthcare [31]. In the Cavallucci 2026 oncology cohort, 82.9% accepted AI with physician control, while only 8.5% accepted AI-driven care without it; 73.5% still held physicians responsible even with AI support [26]. In the Pesapane 2023 mammography cohort, 88% with AI knowledge viewed it positively, but 94% wanted radiologists to finalize reports, and only 77% accepted AI as a second reader [30]. Older Italian women over 60 with prior screenings were more likely to accept AI under radiologist supervision [30]. This pattern shows patient approval of AI-assisted care with a demand for physician accountability.

The principal patient concerns were grouped into three themes. The first theme is the perceived loss of the human aspect of care: in Cavallucci 2026, 63.2% of respondents noted reduced physician contact as a major concern, and 80.3% valued ongoing physician interaction [26]; in Pesapane 2026, 58% at the cancer referral center mentioned reduced personal interaction [31]. The second theme is the reliability and interpretability of AI outputs: 61% of patients in Pesapane 2026 had reliability concerns despite a generally positive view [31], and 52.1% in Cavallucci 2026 were worried about physicians losing clinical decision-making capacity due to AI reliance [21]. The third theme is accountability and liability: 52% of women in Pesapane 2023 believed both the software developer and radiologist should be jointly accountable for AI-related errors [30], aligning with La Regina 2025, where 66–68% attributed responsibility for safe care mainly to physicians [28].

The 2026 survey by Pesapane et al. provides the first Italian evidence on AI disclosure’s impact on patient trust [32]. Six hundred women undergoing mammography were assigned to one of four report scenarios: radiologist-only, AI without flagging, AI-flagged, and AI-flagged with explanation. AI disclosure without context increased anxiety and reduced trust, while disclosure with explanations preserved trust and reduced worry [32]. The authors described AI disclosure as a dilemma between non-disclosure (simple but opaque) and full disclosure (undermines trust if uncontextualized), favoring an intermediate approach [32]. These findings are consistent with the trust-mediated acceptance pattern observed in the cross-sectional surveys [26,30,31] and add experimental evidence that, within the conditions of this single study, the modality of AI communication was associated with patient responses independently of AI involvement.

Two studies explored AI-adjacent data protection, consent, and biobanking, key to patient acceptance of AI-mediated digital health. In Giannella 2024, 1410 of 1454 volunteers and patients consented to biobanking; 99.1% of volunteers and 98.3% of patients approved genetic study use. Only 4.6% of volunteers and 6.6% of patients restricted EU sample sharing, while 7.4% and 10.7% limited extra-European sharing [27]. A significant difference was in result return, with 43% of volunteers and 11.3% of patients preferring no incidental findings (*p* < 0.0001) [27]. In Cavallucci 2026, 76.9% of oncology patients allowed data use for future research, and 60.7% had no data-sharing concerns with other centers [26]. Both studies show broad biobanking and data-sharing acceptance in Italian settings, with more concern for extra-European transfer and result communication.

Mahlknecht 2023 provides unique Italian patient–physician evidence on AI-driven symptom checkers in primary care [29]. In a 2021 Bolzano study, 116 adults used a chatbot for symptoms before seeing their GP, with 122 GP post-visit questionnaires from 10 practices. Patient and physician evaluations differed: 49% of patients were satisfied, versus 27% of physicians; 51% of GP evaluations found chatbot outputs concordant with their clinical appraisals, while 49% did not [29]. Patients liked the ease of use, structure, time-saving, and self-reflection; 47% considered at-home use for initial health appraisals [29]. Physicians noted technical issues and the necessity of physical exams. The study aligns with other findings of moderate patient acceptance under physician oversight and skepticism about AI reliability in unsupervised care.

Synthesizing the seven A1 studies reveals three consistent findings in the Italian AI healthcare evidence base (Table 2). First, AI acceptance as a clinical tool is generally positive (80–96% across studies) but depends on physician oversight and AI transparency [26,30,31,32]. Second, patients mainly worry about dehumanization, AI output reliability, interpretability, and error accountability, with data protection concerns being less significant [26,27,28,30,31]. Third, AI literacy varies by age, education, and nationality, affecting AI care engagement and integration preferences [26,28,30]. The studies are methodologically narrow: cross-sectional surveys at single institutions or networks dominate; Northern and Central Italy provide most data, with Southern Italy and islands under-represented; only one study documents patient experience with current AI tools [29].

### 3.3. Italian Healthcare Professional Perspectives (Class A2)

The 22 studies in Class A2 [33,34,35,36,37,38,39,40,41,42,43,44,45,46,47,48,49,50,51,52,53] span the full range of medical and allied-health professions, from physicians of multiple specialties to nurses, physiotherapists, qualitative researchers, and trainees at residency and student level.

#### 3.3.1. Radiology and Radiation Oncology

Four studies involved Italian radiologists and radiation oncologists [34,37,39,46]. Coppola 2021, the largest study in this group, provided an early national baseline on radiologist attitudes towards AI in an SIRM-member sample [39]. Of respondents, 77% favored AI, 18% were uncertain, and 5% were unfavorable. Perceived advantages included reduced diagnostic errors (73%) and work optimization (67.9%); risks included diminished reputation compared to non-radiologists (60.3%) and increased costs and workload (39%). Most (90.4%) saw regulatory policies as necessary, and 88.9% were not afraid of job loss to AI [39]. The main concern was not job loss but reputation dilution relative to clinicians.

In 2024, Cè surveyed 232 SIRM Lombardy radiologists [37]. Of respondents, 36.2% used AI daily, 38.8% did not, and 25.0% were uncertain. Among users, 30% found AI decisive and 27% ineffective; 85% of non-users saw potential benefits. Overall, 68% were optimistic. Positive opinions were highest in radiologists under 30 (76%) and over 60, while ages 30–60 were more cautious [37]. AI literacy was low: 36% rated knowledge as poor, 43% as barely sufficient.

Carriero 2026 surveyed 4287 early-career radiologists, with 204 valid responses (4.76% response rate) [34]. The study examined work–life balance and career expectations, emphasizing AI training. Most (68.4%) used AI rarely or not at all, 31.4% were self-taught. Lack of AI training was the top challenge (44.6%), followed by lack of mentorship (38.2%) and fellowship programs (35.8%); 48.5% felt “somewhat prepared” for AI changes, and 27.0% unprepared, especially first- and second-year residents (43.4%) [34].

Piras 2025 surveyed 76 Young section radiation oncologists in the Associazione Italiana di Radioterapia ed Oncologia Clinica (yAIRO) network on ChatGPT use [46]. Seventy-three were familiar with ChatGPT, and 71.1% had used it. Of respondents, 40.8% strongly agreed AI could change the medical landscape; 79.1% found AI most useful in research like literature review and protocol drafting; 43.4% used ChatGPT in clinical practice with fair satisfaction (43.2%); 69.7% believed in clinical implementation, though 53.9% feared a negative impact. Main obstacles were medico-legal concerns, lack of clinical evidence, and patient-privacy risks [46].

Across four imaging studies, a recurring pattern shows optimism about AI’s potential and concerns about training, regulatory clarity, and professional identity, stable from the 2021 SIRM baseline to the 2025–2026 surveys.

#### 3.3.2. Specialty-Specific Clinical AI Applications

Eight studies explored AI in clinical specialties beyond radiology [33,36,38,40,43,45,47,52]. Two examined AI in gastroenterological endoscopy: Maida 2025 in adult gastroenterology and Ancona 2026 in pediatric gastroenterology focusing on coeliac-disease endoscopy [33,45]. Rizzo 2026 conducted a national survey on AI/Machine Learning (ML) in clinical microbiology labs, discussing applications, barriers, and ethics [47]. Ciulli 2026 surveyed Tuscan emergency department triage nurses on AI-assisted triage [38]. These surveys show cautious endorsement: respondents see AI’s utility in tasks like lesion detection and triage prioritization but express concerns about accountability, training, and workflow integration.

Three studies addressed AI applications. Danieli 2021 evaluated a conversational AI mental-health agent, providing Italian evidence on AI in psychotherapy [40]. Forte 2025 compared ChatGPT and rheumatologist responses to psoriatic-arthritis patient queries, assessing ChatGPT’s diagnostic accuracy and perceptions of AI communication [43]. Cascella 2024 conducted pre- and post-workshop surveys at the ATLAS 2024 fibromyalgia congress with multidisciplinary participation [36]. The studies show a pattern: engaging with AI in specialty applications shifts concerns from AI replacing clinicians to output reliability, generalizability, and AI–clinician labor division.

Deodato 2025 examines chatbot use in non-physician health professions [42]. Among Italian physiotherapists, 93.3% knew of AI chatbots, but 66.9% never used them clinically. Of those who did, 25.8% had positive experiences. Despite limited use, 78% were positive about future adoption, with 50% seeing chatbots as helpful. Key risks were patient self-diagnosis (84.4%), false information (72.1%), and reduced human interaction (64%). Older physiotherapists with over 21 years of experience used AI chatbots more (odds ratio 5.93; *p* = 0.013) [42], challenging the digital-natives hypothesis and suggesting professional autonomy and clinical confidence influence AI tool integration.

#### 3.3.3. Cross-Specialty AI Knowledge, Attitudes, and Educational Priorities

Seven studies examined AI knowledge and attitudes in various medical specialties [35,42,45,49,51,52,53]. Casà 2021, the earliest, surveyed young Italian physicians on digital competencies and AI readiness during COVID-19 [35]. It documented varied AI competencies, with respondents reporting limited digital-health training, and identified telemedicine, electronic health records, and AI-supported decision support as key areas of anticipated change.

Tozzi 2023 surveyed Italian pediatricians on educational priorities for emerging technologies, using bibliometric analysis and respondent rating to identify priorities; AI was a high-priority but under-trained area [51]. Torricelli 2025 surveyed Northern Italian hospital physicians (response rate 48%): 47% rated their AI knowledge as mild, 30% as poor, and 15% as good, with 98% believing AI will reduce medical errors [49]. Emotional responses included optimism (34%), worry (30%), and enthusiasm (13%), while anxiety was 9%. Women were more pessimistic than men, affecting perceived training impact and professional liability expectations [49]. Anticipated negative outcomes included legal issues, doctor–patient relationship decline, and changes in medical roles.

Cofini 2026 reported the first national application of the I-KAPCAM-AI-Q [53]. Among Italian physicians, 64.8% had basic AI knowledge, and only 18.4% received AI training. Just 21.6% used AI in practice, mainly in diagnostic imaging (35.4% of AI users; 7.7% of the total). Main barriers were lack of training (76.7%) and resistance to change (50.9%). When given a scenario with ChatGPT-generated diagnoses, the agreement with the correct AI diagnosis was 89% (95% CI 86–91%), much higher than with incorrect options, showing physicians can distinguish appropriate AI outputs without prior AI tool use [53].

The “optimism–knowledge gap”, as introduced by Perrella et al. [25] and used here as a descriptive label, denotes the co-occurrence of high stated interest in AI with low self-reported knowledge, limited formal training, and minimal practical exposure within the same respondent group. Perrella 2026 explicitly introduced the regulatory dimension. A national survey on the “human-in-the-loop” framework by the “Garante per la Protezione dei Dati Personali” (Italy’s data protection authority) revealed an “optimism–knowledge gap”: 89.9% of respondents were interested in AI, but practical exposure was low, especially among GPs, 44.1% of whom had never used an AI tool, compared to 34.9% of hospital-based clinicians (χ^2^ = 3.14; *p* = 0.045) [25]. Additionally, 32.6% of GPs understood “some benefits but not the limitations” of AI, posing a regulatory and clinical risk under the Italian human-in-the-loop legal mandate [25].

Vozzi 2026 surveyed Italian neurology residents in a national web-based survey, again documenting limited formal training, mixed but predominantly positive attitudes, and a strong demand for structured AI education in residency [52]. Across the seven cross-specialty studies, three convergent findings emerge: Within the descriptive synthesis, the “optimism–knowledge gap” was operationalized through three empirical indicators jointly present in a given respondent group: (i) self-reported interest in or positive attitude towards AI in a majority of respondents, (ii) self-assessed AI knowledge rated as inadequate by a majority of the same respondents, and (iii) low formal training exposure or low practical use of AI tools in the same group. The label was applied where all three indicators co-occurred within a study or were aggregable across studies of the same population stratum. AI knowledge is consistently self-assessed as inadequate (typically below 20% for self-reported good knowledge); training opportunities are perceived as scarce (gaps reported in 44–77% of respondents across studies); and attitudes are predominantly positive in principle but conditional on the resolution of regulatory, training, and accountability gaps. The recurrent “optimism–knowledge gap” motif, named explicitly by Perrella 2026 [25] but visible across all seven studies, characterizes the Italian cross-specialty professional evidence base on AI in healthcare.

#### 3.3.4. Health-Professional Students and Qualitative Inquiry

Three studies used designs complementing cross-sectional, professional surveys [41,48,50]. Tortella 2025 surveyed Italian physiotherapy students on AI chatbot knowledge and use [50]. Supported by the Health Service of South Tyrol in Bolzano, it documented student exposure, attitudes, and educational expectations, aligning with Deodato 2025 [42]: high awareness, limited use, and strong AI training support. Two qualitative studies add interpretive depth.

Rossero & Lombi 2026 conducted interviews with Italian surgeons and radiologists on trust, risk, and professional boundaries in robotic and AI care [48]. The analysis identified “recalibrating expectations”: clinicians differentiated between supportive and displacing automation, viewing trust as active negotiation. AI and clinician work boundaries were renegotiated case by case, influenced by AI output visibility, clinical situation predictability, and accountability frameworks [48].

Dellafiore 2026 conducted a reflexive thematic analysis with expert qualitative researchers on AI use in qualitative inquiry [41]. Though not a clinical AI tool, it was included as participants were healthcare professionals, mainly nursing and allied-health researchers. Thematic findings on trust in AI outputs, AI-mediated coding reliability, and ethical human–AI collaboration link to trust-and-accountability themes in the A2 corpus [41]. Across three studies, qualitative inquiry highlights complexities of trust, practice, and professional identity that complement quantitative evidence.

#### 3.3.5. Synthesis of the A2 Evidence

Synthesizing 22 A2 studies reveals 4 consistent findings on AI in Italian healthcare (Table 3). First, professional acceptance is broadly positive (68–98%) but depends on training, regulatory clarity, and autonomy [25,34,37,39,49,53]. Second, AI knowledge and training are limited across specialties, with formal training reported by 17–18% in recent surveys and lack of training as the main adoption barrier for 45–77% [25,34,53]. Third, concerns focus on professional identity, doctor–patient relationship, and medico-legal issues, not job replacement, feared by less than 12% [39,49]. Fourth, an “optimism–knowledge gap” exists, with high enthusiasm but low practical exposure, especially in primary care [25].

### 3.4. Italian Acceptance Instrument Validation Studies (Class A3)

Three studies in the corpus are psychometric validations of acceptance instruments adapted or developed for Italian-speaking populations [54,55,56]. Together, they make available, for the first time, three culturally adapted measurement tools for AI-acceptance research in Italy: a domain-specific instrument for healthcare providers (I-KAPCAM-AI-Q), a multidimensional cross-domain instrument adapted from international technology-acceptance theory (AIDUA-IT), and a general attitudinal instrument with a positive–negative bipolar structure (GAAIS-IT). Each instrument addresses a different theoretical construct and a different target population, and the three studies should therefore be read as complementary rather than competing.

Cofini 2025 developed and validated the Italian Knowledge, Attitudes, Practices and Clinical Application of Medical AI Questionnaire (I-KAPCAM-AI-Q) [56]. The 29-item tool includes one universal and six specialty-specific clinical scenarios to assess healthcare providers’ AI readiness. Validation involved expert review, face-validity assessment, technical testing, and pilot testing. The instrument showed strong content validity (Scale-level Content Validity Index/Averaging method [S-CVI/Ave] = 0.98), acceptable internal consistency (Cronbach’s α = 0.7481; Kuder–Richardson 21 = 0.832), and convergent evidence. In the pilot, 17% received digital training, and 91% agreed with AI diagnoses; residents showed more interest than specialists in technical support (58.3% vs. 42.0%; *p* = 0.021) and evidence-based validation (61.2% vs. 47.0%; *p* = 0.043) [56]. Cofini 2026 (Section 3.3.3) [53] reports the first national application, confirming its ability to differentiate AI users from non-users.

Cavasin 2026 reported the adaptation and preliminary validation of the Italian version of the Artificially Intelligent Device Use Acceptance scale (AIDUA-IT) [54]. The original AIDUA model defines AI acceptance with eight factors: Social Influence, Hedonic Motivation, Anthropomorphism, Performance Expectancy, Effort Expectancy, Emotion, Intention/Willingness to Use, and Objection to Use. Validation followed COSMIN and STROBE guidelines through a two-phase design (translation, expert review, cognitive debriefing, then structural-validity, internal-consistency, convergent, and discriminant validity, and test–retest assessments). The eight-factor model fit well (CFI = 0.984; TLI = 0.981; RMSEA = 0.041; SRMR = 0.056), with strong loadings (β 0.64–0.96) and good internal consistency (Cronbach’s α and McDonald’s ω 0.82–0.90). Convergent and discriminant validity were supported, and test–retest reliability over two weeks was good to excellent (Intraclass Correlation Coefficient 0.81–0.90) [54]. The sample had a subject-to-item ratio of 4.5:1, below the 10:1 threshold for factor analysis with eight dimensions, and AIDUA-IT is framed as preliminary, needing confirmation in larger samples.

Cicero 2025 validated the Italian version of the General Attitudes towards Artificial Intelligence Scale (GAAIS-IT) through two studies on independent samples [55]. The GAAIS has 20 items measuring 2 factors: positive (12 items on perceived opportunities and benefits) and negative (8 items on concerns). Study 1’s confirmatory factor analysis supported a two-factor model with correlated residuals (items 3/6 on AI ethics; items 13/16 on AI superiority) showing good fit (χ^2^/df = 1.97; CFI = 0.907; RMSEA = 0.064; SRMR = 0.070), better than a unidimensional model (CFI = 0.591). Internal consistency was good (Cronbach’s α = 0.85; McDonald’s ω = 0.86). Study 2 evaluated validity: the positive factor correlated positively with AI attitudes (r = 0.57; *p* < 0.01), the negative factor correlated negatively (r = −0.31; *p* < 0.05). In regression, the positive factor explained 47.3% of AI-use intention variance (β = 0.688; *p* < 0.001), the negative factor 4.0% (β = −0.200; *p* = 0.008), supporting both as predictors [55]. The authors noted the two-factor structure might reflect item-wording effects and suggested further testing.

Synthesizing the three A3 studies reveals three key observations for the Italian evidence base (Table 4). First, before 2025, no validated Italian-language acceptance instrument existed for AI in healthcare or public services; these studies fill this gap (provider readiness, cross-domain acceptance, and attitudinal orientation). Second, all instruments are in early validation: Cofini 2025 is a pilot [56], Cavasin 2026 is preliminary [54], and Cicero 2025 notes issues with factorial invariance [55]; larger, diverse validation samples and longitudinal evidence are needed. Third, the instruments measure different constructs: I-KAPCAM-AI-Q assesses physician readiness, AIDUA-IT uses a behavioral-intention model, and GAAIS-IT measures attitudinal orientation in the general population.

### 3.5. Italian Mixed-Population Evidence (Class B)

One study combined Italian and Dutch participants to assess assistive robotics for older adults [57]. Fiorini 2021, part of the European Agile Co-Creation for Robots and Aging (ACCRA) project, used semi-structured interviews at Italian and Dutch sites to collect mobility needs and attitudes toward an assistive robot. The study was included because assistive robotics involves AI-based perception and decisional functions and provides unique Italian data on technology acceptance among older adults and caregivers.

In Italy, eight of ten older adults had positive perceptions of the robot; among caregivers, 22 of 30 were positive. Caregivers noted mobility dysfunctions and fall risk as key areas for robotic assistance, with one highlighting fall-risk prediction through gait monitoring [57]. The Dutch sample was more cautious, with 5 of 10 older adults positive and caregivers split between neutral and negative views. The comparison shows a more positive Italian attitude toward assistive robotics, especially among caregivers, while concerns in both countries included loss of human contact and technical reliability, echoing trust-and-accountability themes across the A1 and A2 corpus.

### 3.6. International Comparator Studies with Italian Sub-Samples (Class C)

Two studies within the INCISIVE EU Horizon 2020 project, a multinational research consortium developing an AI-based toolbox and a federated imaging repository for cancer diagnosis and follow-up, provide European evidence on AI in cancer care, with significant Italian participation through the University of Naples Federico II [58,59]. Hesso 2023 combined an online survey of 95 oncology professionals across seven partner countries with semi-structured interviews of 27 professionals [58]; Hesso 2024 used email-based qualitative interviews with ten oncology specialists to map cancer care pathways and AI’s potential role [59]. Italy contributed 26 of the 95 survey respondents (27%) and 7 of the 27 interviewees (26%) in Hesso 2023 [58], making it the second-largest national contingent after Greece, and one of the ten national experts in Hesso 2024 [59].

Oncology professionals expressed positive expectations regarding AI’s contribution to cancer imaging and streamlining diagnostic and therapeutic pathways. Barriers included lack of explainability of AI outputs, with participants rejecting black-box tools and requiring interpretability; data quality, harmonization, and availability for AI training; and lack of structured education for AI engagement [58]. Facilitators included evidence-based validation of AI accuracy, user-friendly tool design, and patient-awareness initiatives. Hesso 2024 noted that, except for the UK, none of the countries had national data on diagnostic and therapeutic delays in cancer care, a barrier AI-assisted pathways could address but that limits evaluative infrastructure [59].

Italian-specific findings align with patterns in the A2 corpus: cautious optimism about AI utility, acceptance contingent on training and regulatory clarity, and concern with explainability and accountability rather than workforce displacement. The Class C evidence’s value lies in situating Italian oncology professionals’ views within a European frame, showing that themes in Italian studies reflect broader European patterns shared by professionals in different healthcare systems.

## 4. Discussion

This scoping review mapped the empirical evidence on attitudes, acceptance, and perceptions of artificial intelligence in healthcare in Italy and identified three convergent patterns. Across patient and general-population studies, AI acceptance ranged from 80% to 96%, conditional on physician oversight and on the modality of AI disclosure [26,30,31,32]. Across healthcare professionals, acceptance ranged from 68% to 98%, conditional on training, regulatory clarity, and preserved professional autonomy. Formal AI training was reported by only 17–18% of physicians and was described as the principal adoption barrier by 45–77% of respondents [25,34,39,53]. Three Italian-validated measurement instruments (I-KAPCAM-AI-Q, AIDUA-IT, GAAIS-IT) became available between 2025 and 2026, addressing complementary constructs but all at preliminary validation stages [54,55,56].

These patterns converge with European evidence: a global integrative review of hospital-based clinicians documented similar conditional optimism with concerns about autonomy and workflow [7]; a UK survey reported 79% expecting AI utility against 80% fearing privacy problems [8]; over 1000 Portuguese physicians described curriculum gaps as the principal adoption barrier [9]; and a German qualitative study identified human oversight, robust evidence, data protection, and clear legal standards as preconditions for physician acceptance [60]. The attitudinal pattern observed in Italian respondents—including the optimism–knowledge gap as operationalized in Section 3.3.3, the conditional structure of acceptance, and trust-mediated responses to AI disclosure—is broadly consistent with that reported in the United Kingdom [8], Portugal [9], and Germany [60] and is therefore not idiosyncratic to Italy. What distinguishes the Italian evidence base is not the attitudinal pattern itself but the regulatory architecture within which it is situated. The human-in-the-loop mandate of Law 132/2025 [17] and the “Garante” framework [16,45] transform physician oversight from a clinical preference into a binding requirement, with direct consequences for the level of clinician competence and population literacy needed for compliant AI deployment.

The principal methodological gap identified in this review concerns the absence of probabilistic, population-representative evidence on AI attitudes in Italy. The seven Class A1 studies are exclusively cross-sectional convenience samples drawn from single institutions or specialized clinical populations; none use a probabilistic sampling frame stratified to a national or regional reference population, and Northern and Central Italy dominate the geographical distribution while Southern Italy and the islands remain under-represented [26,27,28,29,30,31,32]. This design profile is not confined to the patient and general-population stratum: of the 35 included studies, 23 are cross-sectional surveys, the great majority recruit through professional-society mailing lists, single-institution rosters, or other non-probabilistic channels, and the geographical distribution is concentrated in Northern and Central Italy across all population strata. The identified themes—conditional acceptance, the optimism–knowledge gap, and trust-mediated responses—should accordingly be read as patterns observable across a methodologically narrow evidence base rather than as estimates generalizable to the Italian healthcare system. These features jointly limit the inferential reach of the existing literature and constrain its use for healthcare-policy formulation at a population level.

In the research program within which the present scoping review is embedded, this gap is addressed by a population survey commissioned by the authors’ institute and conducted by the Provincial Institute of Statistics of the Autonomous Province of Bolzano-South Tyrol (ASTAT). The survey forms part of the probabilistic panel “So denkt Südtirol/Così pensa l’Alto Adige” (February 2026 wave). Stratification was by sex, territory and age class, with calibration on sex, age, area of residence, and educational level (*n* = 901; response rate 75%). The descriptive first-wave results have been published as an official statistical bulletin [61]; an analytical study with explicit comparison to European cohorts and to the Italian-study findings synthesized here is in preparation.

A second methodological gap concerns the absence of multilingual evidence within the Italian context, which limits comparative analysis across language groups in regions such as South Tyrol, Friuli-Venezia Giulia, and Valle d’Aosta. Building on the Class A2 findings of this review, an analogous survey of healthcare professionals in South Tyrol is in preparation. Instrument selection draws on the three Italian-validated tools mapped in Class A3 (I-KAPCAM-AI-Q for provider readiness [56], AIDUA-IT for behavioral intention [54], GAAIS-IT for general attitudinal orientation [55]), with culturally adapted bilingual versions to enable comparative analysis across the German- and Italian-speaking professional communities.

**Figure 2:**
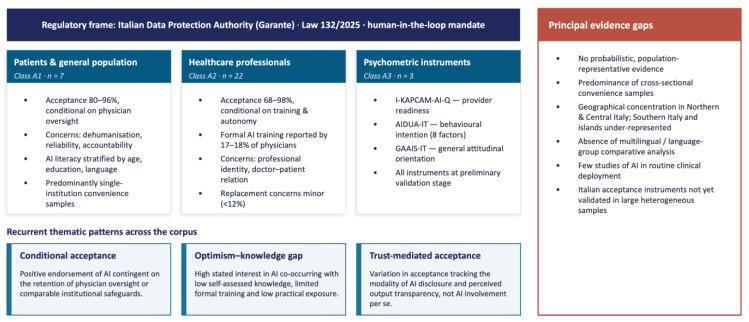
Conceptual summary of the scoping review. The figure organizes the synthesis along three axes: the population strata identified in the corpus (Italian patients and general population, Class A1; healthcare professionals, Class A2; psychometric validation studies, Class A3), the recurrent thematic patterns observed across studies (conditional acceptance, optimism–knowledge gap, trust-mediated acceptance), and the regulatory frame within which Italian AI deployment is situated (Garante framework and Law 132/2025). The principal evidence gaps that motivate the future-research agenda are summarized on the right.

Three implications follow for research and policy. First, structured AI training in residency and continuing professional development should be a priority across Italian health-professional curricula, given the consistency with which trainees and early-career professionals across the corpus identify training gaps as the principal adoption barrier [34,46,52]. Second, transparent AI-disclosure practices in clinical communication are required, in line with the experimental evidence that the modality of disclosure—rather than AI involvement per se—shapes patient trust [32]. Third, the Italian regulatory framework and its human-in-the-loop architecture should be supported by empirical evidence on practical implementation in primary and hospital care, particularly in regions with multilingual or institutionally heterogeneous configurations. In terms of study design, four priorities are identifiable from the gaps mapped in this review: probabilistic population-representative surveys with sampling frames stratified by region and language group to address the inferential limitations of the current convenience-based evidence; longitudinal designs to characterize the trajectory of acceptance as practical exposure increases; bilingual or multilingual instrument adaptations to enable comparative analysis across language groups in regions such as South Tyrol; and implementation studies tracking acceptance and concordance in clinical settings where AI tools are actively deployed, rather than hypothetical-scenario surveys. A conceptual summary of the synthesis is provided in Figure 2.

Methodological limitations of this scoping review are acknowledged. First, the protocol was registered retrospectively on the Open Science Framework after execution of the systematic search. Second, while inter-rater reliability was estimated through independent re-screening of a 20% random sample by a second reviewer with high agreement, the remaining 80% of records were screened by the lead reviewer alone. Third, the grey literature was not searched systematically; sources such as the Italian National Institute of Statistics (ISTAT), the Ministry of Health, regional health authorities, and professional associations were consulted only ad hoc. Fourth, the included Italian evidence is methodologically narrow—predominantly cross-sectional, predominantly convenience-based, and geographically concentrated in Northern and Central Italy with limited representation of Southern Italy and the islands—which constrains the generalizability of the synthesized patterns to the Italian healthcare system.

## 5. Conclusions

This scoping review mapped empirical evidence on attitudes, acceptance, and perceptions of artificial intelligence in Italian healthcare across 35 studies from 2018 to April 2026, with nearly two-thirds published in the last 18 months. Three patterns emerged: AI acceptance among Italian patients was 80% to 96%, contingent on physician oversight, AI transparency, and disclosure; among healthcare professionals, it was 68% to 98%, dependent on training, regulatory clarity, and professional autonomy, with only 17–18% having formal AI training, identified as the main adoption barrier. An optimism–knowledge gap, with high enthusiasm but low practical exposure, especially in primary care, characterizes the Italian professional evidence base. Three Italian-validated acceptance instruments (I-KAPCAM-AI-Q, AIDUA-IT, and GAAIS-IT) emerged between 2025 and 2026, addressing complementary constructs but at preliminary validation stages. Principal gaps include the absence of probabilistic, population-representative evidence, geographical concentration in Northern and Central Italy, and lack of multilingual comparative analysis. Future research should prioritize representative surveys, multilingual designs in regions like South Tyrol, and structured AI training in residency and professional development.

## Figures and Tables

**Table 1:** Characteristics of the 35 empirical studies included in the scoping review, ordered by population and first author.

First Author, Year	Study Design	Population	Sample Size ^1^	Setting/Region	AI Application Domain
Italian general population and patients (Class A1)					
Cavallucci 2026 [26]	Cross-sectional survey	Oncology patients (IRCCS IRST)	117	Regional (Emilia-Romagna)	AI attitudes/data protection
Giannella 2024 [27]	Cross-sectional	Volunteers, neurological patients (IRCCS S. Lucia)	1454	Regional (Rome)	Biobanking/data privacy
La Regina 2025 [28]	Representative panel survey	Italian general population (DOXA)	1200	National	Patient safety culture (AI sub-component)
Mahlknecht 2023 [29]	Mixed-methods feasibility	Patients and GPs	10 GPs, 116 pts.	Regional (South Tyrol/Bolzano)	Symptom checker/primary care
Pesapane 2023 [30]	Prospective survey	Women in mammography screening (IEO)	800	Regional (Milan)	Diagnostic imaging/screening
Pesapane 2026 [31]	Cross-sectional patient survey	Cancer referral center patients	240	Regional (Milan)	AI in medicine and radiology
Pesapane 2026 [32]	Randomized survey study	Women in mammography screening	600	Regional (Milan)	AI disclosure in radiology reports
Italian healthcare professionals (Class A2)					
Ancona 2026 [33]	Cross-sectional survey	Pediatric gastroenterology centers (SIGENP)	38	National	AI in endoscopy/coeliac disease
Carriero 2026 [34]	Cross-sectional survey	Radiology residents and young radiologists (SIRM Young)	204	National	AI in radiology training/practice
Casà 2021 [35]	Cross-sectional survey	Young Italian physicians (SIGM)	362	National	Digital competencies/AI readiness
Cascella 2024 [36]	Cross-sectional survey	Multidisciplinary clinicians (rheumatologists, physiatrists, anesthetists, neurologists) pre-/post-workshop	26	National	AI in fibromyalgia management
Cè 2024 [37]	Cross-sectional survey	Radiologists	232	Regional (Lombardy)	AI integration/readiness
Ciulli 2026 [38]	Cross-sectional survey	Triage nurses (emergency dept)	84	Regional (Tuscany)	AI in triage
Cofini 2026 [53]	Cross-sectional survey	Physicians (I-KAPCAM-AI-Q)	587	National	AI knowledge and concordance
Coppola 2021 [39]	Cross-sectional survey	Radiologists (SIRM)	1032	National	Diagnostic imaging
Danieli 2021 [40]	Feasibility study	Clinicians; mental-health AI agent	21	National	Conversational AI/mental health
Dellafiore 2026 [41]	Reflexive thematic analysis	Expert qualitative researchers	14	National	AI in qualitative inquiry
Deodato 2025 [42]	Cross-sectional survey	Physiotherapists (AIFI)	415	National	AI chatbots in clinical practice
Forte 2025 [43]	Cross-sectional survey	Rheumatologists and PsA patients	76 pts; 12 specialists	National	Generative AI/ChatGPT comparison
Giansanti 2025 [44]	Cross-sectional survey	Digital cytology professionals (ISS)	150	National	Digital pathology/cytology
Maida 2025 [45]	Cross-sectional survey	Gastroenterologists	150	National	AI in endoscopy
Perrella 2026 [25]	Cross-sectional survey	Clinicians (“Garante” framework)	362	National	Optimism–knowledge gap
Piras 2025 [46]	Cross-sectional survey	Young radiation oncologists (yAIRO)	76	National	ChatGPT use in oncology
Rizzo 2026 [47]	Cross-sectional survey	Clinical microbiology laboratories (AMCLI/GLAIMAL)	163	National	AI/ML in microbiology
Rossero 2026 [48]	Qualitative interviews	Surgeons and radiologists	24	National	Robotic and AI-enhanced care
Torricelli 2025 [49]	Cross-sectional survey	Hospital physicians	176	Regional (Northern Italy)	AI fears and expectations
Tortella 2025 [50]	Cross-sectional survey	Physiotherapy students	589	National	AI chatbots/education
Tozzi 2023 [51]	Cross-sectional survey	Pediatricians (Bambino Gesù)	1540	National	Educational priorities/emerging tech
Vozzi 2026 [52]	Cross-sectional web-based survey	Neurology residents	173	National	AI training/neurology
Italian acceptance instrument validation studies (Class A3)					
Cavasin 2026 [54]	Psychometric validation	Italian-speaking adults (CFA)	140	Regional (Padova)	Validation: AIDUA-IT
Cicero 2025 [55]	Psychometric validation	General Italian-speaking adults	236	National	Validation: GAAIS-IT
Cofini 2025 [56]	Psychometric validation	Physicians (residents + specialists) pilot study	203	National	Validation: I-KAPCAM-AI-Q
Italian study with mixed populations (Class B)					
Fiorini 2021 [57]	Qualitative needs study	Older adults and carers (ACCRA)	34 caregivers, 20 pts.	Italy + The Netherlands	Assistive robotics
International comparator studies (Class C)					
Hesso 2023 [58]	Mixed-methods study	Oncology HCPs (INCISIVE-EU)	95 (survey), 27 (interview)	Italy + 6 European countries	Cancer-care AI
Hesso 2024 [59]	E-mail interviews	Oncology specialists (INCISIVE-EU)	10	Italy + 7 European countries	Cancer-care AI

^1^ Sample size *n* is given for the eligible respondent group analyzed. Abbreviations: ACCRA, Agile Co-Creation for Robots and Aging; AI, artificial intelligence; AIDUA-IT, Italian version of the Artificially Intelligent Device Use Acceptance scale; AIFI, Associazione Italiana di Fisioterapia; AMCLI, Associazione Microbiologi Clinici Italiani; CFA, confirmatory factor analysis; ATLAS, Annual Thinking Lab on Fibromyalgia Syndrome; DOXA, Italian polling institute (BVA-Doxa); GAAIS-IT, Italian version of the General Attitudes towards Artificial Intelligence Scale; GLAIMAL, Gruppo di Lavoro AI/ML in Microbiologia; GP, general practitioner; HCP, healthcare professional; IEO, European Institute of Oncology; I-KAPCAM-AI-Q, Italian Knowledge, Attitudes, Practices and Clinical Application of Medical AI Questionnaire; IRCCS, Istituto di Ricovero e Cura a Carattere Scientifico; IRST, Istituto Romagnolo per lo Studio dei Tumori; ISS, Istituto Superiore di Sanità; ML, machine learning; PsA, psoriatic arthritis; SIGENP, Società Italiana di Gastroenterologia, Epatologia e Nutrizione Pediatrica; SIGM, Segretariato Italiano Giovani Medici; SIRM, Società Italiana di Radiologia Medica e Interventistica; yAIRO, Associazione Italiana di Radioterapia ed Oncologia Clinica.

**Table 2:** Synthesis of main findings on Italian patient and general-population perspectives (Class A1).

Population Focus	Studies (*n*)	Convergent Findings	Predominant Concerns	Evidence Gaps	References
Italian patients and general population	7	High AI acceptance (80–96%) conditional on physician oversight; AI literacy stratified by age and education; modality of AI disclosure shapes patient trust	Loss of the human aspect of care; reliability and interpretability of AI outputs; accountability for AI-related errors	Predominantly single-institution surveys; Southern Italy and islands under-represented; only one study with a deployed AI tool	[26,27,28,29,30,31,32]

**Table 3:** Synthesis of main findings on Italian healthcare-professional perspectives (Class A2).

Population Focus	Studies (*n*)	Convergent Findings	Predominant Concerns	Evidence Gaps	References
Italian healthcare professionals	22	Broadly positive professional acceptance (68–98%) conditional on training, regulation, and professional autonomy; recurrent “optimism–knowledge gap”; high agreement with appropriate AI outputs (89%) even among non-users	Professional identity and reputation; doctor–patient relationship; medico-legal accountability; replacement concerns minor (<12%)	Formal AI training reported by 17–18% of physicians; lack of training is the main adoption barrier; few studies document AI use in routine workflows	[25,33,34,35,36,37,38,39,40,41,42,43,44,45,46,47,48,49,50,51,52,53]

**Table 4:** Synthesis of main findings on Italian acceptance-instrument validation studies (Class A3).

Population Focus	Studies (*n*)	Convergent Findings	Predominant Concerns	Evidence Gaps	References
Italian psychometric validations of AI-acceptance instruments	3	Three culturally adapted instruments now available with complementary constructs: I-KAPCAM-AI-Q (provider readiness), AIDUA-IT (behavioral intention), GAAIS-IT (general attitudes)	All instruments at early validation stages; samples below conventional thresholds for confirmatory factor analysis; potential item-wording effects in the bipolar scale	Larger and more heterogeneous validation samples; tests of factorial invariance; longitudinal evidence; cross-instrument concurrent validation	[54,55,56]

## Data Availability

No new data were created or analyzed in this study. All studies included in this scoping review are publicly available peer-reviewed publications cited in the reference list. The full list of included and excluded records, with eligibility decisions and reasons for exclusion, is provided in Supplementary Table S1. The protocol of this scoping review is publicly available on the Open Science Framework (https://doi.org/10.17605/OSF.IO/TZRVF). The PubMed and Embase search strings are reported verbatim in Section 2.5 of this manuscript and can be reproduced through the respective databases.

## Supplementary Materials

The following supporting information can be downloaded at: https://www.mdpi.com/article/10.3390/medsci14020276/s1, Table S1: PRISMA-ScR Checklist; Table S2: Full-text screening decisions and reasons for all 106 records advanced to the eligibility stage.

## Abbreviations

The following abbreviations are used in this manuscript:

ACCRA	Agile Co-Creation for Robots and Aging
AGENAS	Agenzia Nazionale per i Servizi Sanitari Regionali
AI	Artificial Intelligence
AIDUA	Artificially Intelligent Device Use Acceptance
AIFI	Associazione Italiana di Fisioterapia
AMCLI	Associazione Microbiologi Clinici Italiani
ASTAT	Provincial Institute of Statistics of Bolzano-South Tyrol
ATLAS	Annual Thinking Lab on Fibromyalgia Syndrome
CFA	Confirmatory Factor Analysis
CFI	Comparative Fit Index
CI	Confidence Interval
DOXA	Italian polling institute (BVA-Doxa)
EU	European Union
GAAIS	General Attitudes towards Artificial Intelligence Scale
GDPR	General Data Protection Regulation
GLAIMAL	Gruppo di Lavoro AI/ML in Microbiologia
GP	General Practitioner
HCP	Healthcare Professional
I-KAPCAM-AI-Q	Italian Knowledge, Attitudes, Practices and Clinical Application of Medical AI Questionnaire
IEO	European Institute of Oncology
IRCCS	Istituto di Ricovero e Cura a Carattere Scientifico
IRST	Istituto Romagnolo per lo Studio dei Tumori
ISS	Istituto Superiore di Sanità
ISTAT	Italian National Institute of Statistics
JBI	Joana Briggs Institute
MeSH	Medical Subject Heading
ML	Machine Learning
OR	Odds Ratio
PCC	Population–Concept–Context
PNRR	National Recovery and Resilience Plan
PRISMA-ScR	Preferred Reporting Items for Systematic reviews and Meta-Analyses extension for Scoping Reviews
PsA	Psoriatic Arthritis
RMSEA	Root Mean Square Error of Approximation
SABES/ASDAA	Südtiroler Sanitätsbetrieb/Azienda Sanitaria dell’Alto Adige
S-CVI	Scale-level Content Validity Index
S-CVI/Ave	Scale-level Content Validity Index/Averaging method
SIGENP	Società Italiana di Gastroenterologia, Epatologia e Nutrizione Pediatr
SIGM	Segretariato Italiano Giovani Medici
SIRM	Società Italiana di Radiologia Medica e Interventistica
SRMR	Standardized Root Mean Square Residual
yAIRO	Associazione Italiana di Radioterapia ed Oncologia Clinica
