# Supplementary material for: Conditional Acceptance and the Optimism–Knowledge Gap: A Scoping Review of Attitudes and Perceptions of Artificial Intelligence in Healthcare in Italy
Source: Med Sci (Basel). 2026 May 28;14(2):276. doi: 10.3390/medsci14020276 (PMC13303294; doi:10.3390/medsci14020276)
Supplement: Supplementary file 1 [file medsci-14-00276-s001.zip › medsci-4353352-supplementary.pdf]

Supplementary Materials

Table S1. PRISMA-ScR Checklist

Section	Item	PRISMA-ScR Checklist Item *	Reported on page
		TITLE	
Title	1	Identify the report as a scoping review.	Title page (p. 1)
		ABSTRACT	
Structured summary	2	Provide a structured summary that includes (as applicable) background, objectives, eligibility criteria, sources of evidence, charting methods, results, and conclusions that relate to the review questions and objectives.	Abstract (p. 1)
		INTRODUCTION	
Rationale	3	Describe the rationale for the review in the context of what is already known. Explain why the review questions/objectives lend themselves to a scoping review approach.	Section 1, Introduction (pp. 1–3)
Objectives	4	Provide an explicit statement of the questions and objectives being addressed with reference to their key elements (e.g., population or participants, concepts, and context) or other relevant key elements used to conceptualize the review questions and/or objectives.	Section 1, Introduction (p. 3); Section 2.2, Review Questions (p. 3)
		METHODS	
Protocol and registration	5	Indicate whether a review protocol exists; state if and where it can be accessed (e.g., a Web address); and if available, provide registration information, including the registration number.	Section 2.1, Study Design and Reporting Framework (p. 3); OSF DOI: 10.17605/OSF.IO/TZRVF
Eligibility criteria	6	Specify characteristics of the sources of evidence used as eligibility criteria (e.g., years considered, language, and publication status), and provide a rationale.	Section 2.3, Eligibility Criteria (p. 3)
Information sources	7	Describe all information sources in the search (e.g., databases with dates of coverage and contact with authors to identify additional sources), as well as the date the most recent search was executed.	Section 2.4, Information Sources (p. 3); Section 2.5, Search Strategy (pp. 4–5)
Search	8	Present the full electronic search strategy for at least 1 database, including any limits used, such that it could be repeated.	Section 2.5.1, PubMed; Section 2.5.2, Embase; Section 2.5.3, Consensus Pro (pp. 4–5); Supplementary Material S2 (verbatim search strings)
Selection of sources of evidence	9	State the process for selecting sources of evidence (i.e., screening and eligibility) included in the scoping review.	Section 2.6, Selection of Sources of Evidence (p. 5); Figure 1 (PRISMA flow diagram)
Data charting process	10	Describe the methods of charting data from the included sources of evidence (e.g., calibrated forms or forms that have been tested by the team before their use, and whether data charting was done	Section 2.7, Data Charting Process (p. 5)

Section	Item	PRISMA-ScR Checklist Item *	Reported on page
		independently or in duplicate) and any processes for obtaining and confirming data from investigators.	
Data items	11	List and define all variables for which data were sought and any assumptions and simplifications made.	Section 2.7, Data Charting Process (p. 5)
Critical appraisal of individual sources of evidence	12	If done, provide a rationale for conducting a critical appraisal of included sources of evidence; describe the methods used and how this information was used in any data synthesis (if appropriate).	Section 2.8, Synthesis of Results (p. 6) — no critical appraisal conducted, in line with JBI scoping review methodology
Synthesis of results	13	Describe the methods of handling and summarizing the data that were charted.	Section 2.8, Synthesis of Results (p. 6)
		RESULTS	
Selection of sources of evidence	14	Give numbers of sources of evidence screened, assessed for eligibility, and included in the review, with reasons for exclusions at each stage, ideally using a flow diagram.	Section 3.1, Selection of Sources of Evidence and Characteristics of Included Studies (pp. 6–10); Figure 1; Supplementary Table S1 (all 107 records with eligibility decisions)
Characteristics of sources of evidence	15	For each source of evidence, present characteristics for which data were charted and provide the citations.	Section 3.1 (pp. 6–10); Table 1 (Characteristics of included studies)
Critical appraisal within sources of evidence	16	If done, present data on critical appraisal of included sources of evidence (see item 12).	Not applicable — no critical appraisal conducted (see item 12)
Results of individual sources of evidence	17	For each included source of evidence, present the relevant data that were charted that relate to the review questions and objectives.	Section 3.2, Italian General-Population and Patient Perspectives (Class A1) (pp. 11–12); Section 3.3, Italian Healthcare Professional Perspectives (Class A2) (pp. 13–15); Section 3.4, Italian Acceptance Instrument Validation Studies (Class A3) (pp. 16–17); Section 3.5, Italian Mixed-Population Evidence (Class B) (p. 17); Section 3.6, International Comparator Studies with Italian Sub-Samples (Class C) (pp. 17–18)
Synthesis of results	18	Summarize and/or present the charting results as they relate to the review questions and objectives.	Section 3.2–3.6 (pp. 11–18); Tables 2a, 2b, 2c (Synthesis of main

Section	Item	PRISMA-ScR Checklist Item *	Reported on page
			findings for Classes A1, A2, A3)
		DISCUSSION	
Summary of evidence	19	Summarize the main results (including an overview of concepts, themes, and types of evidence available), link to the review questions and objectives, and consider the relevance to key groups.	Section 4, Discussion (pp. 18–19)
Limitations	20	Discuss the limitations of the scoping review process.	Section 4.2, Limitations (p. 19)
Conclusions	21	Provide a general interpretation of the results with respect to the review questions and objectives, as well as potential implications and/or next steps.	Section 5, Conclusions (pp. 19–20)
		FUNDING	
Funding	22	Describe sources of funding for the included sources of evidence, as well as sources of funding for the scoping review. Describe the role of the funders of the scoping review.	Funding statement (p. 20)

* From Tricco AC, Lillie E, Zarin W, O'Brien KK, Colquhoun H, Levac D, et al. PRISMA Extension for Scoping Reviews (PRISMA-ScR): Checklist and Explanation. Ann Intern Med. 2018;169:467–473. Abbreviations: JBI, Joanna Briggs Institute; OSF, Open Science Framework.

Table S2. Full-text screening decisions and reasons for all 106 records advanced to the eligibility stage.

First author, year [Reference]	Decision	Reason / Note on inclusion or exclusion
Álvarez Sánchez-Bayuela 2024 [62]	Excluded	MammoWave multicentre microwave imaging protocol; tool-performance validation without acceptance assessment
Amabili 2025 [63]	Excluded	engAGE study protocol on social robot intervention; no results reported at the time of screening
Ancona 2026 [33]	Included	Nationwide SIGENP survey of Italian paediatric gastroenterology centres (n = 38) including AI adoption section
Antón-Rodríguez 2026 [64]	Excluded	Spanish concept analysis of accompaniment; AI used only as methodological tool for everyday-language identification
Arcà 2025 [65]	Excluded	International conference abstract on AI in pricing and reimbursement across global healthcare archetypes; HTA-policy review without primary empirical data on attitudes, acceptance, or perceptions of AI in healthcare
Arzilli 2024 [66]	Excluded	Italian PRISMA-ScR-compliant scoping review on AI/ML for healthcare-associated infection surveillance; review without primary empirical data on attitudes, acceptance, or perceptions of AI in healthcare
Baglivo 2025 [67]	Excluded	Commentary on SIIAM vision for AI in the Italian National Health Service without empirical component

First author, year [Reference]	Decision	Reason / Note on inclusion or exclusion
Baglivo 2025 [68]	Excluded	Editorial on the SIIAM Annual Meeting; programme paper without empirical component
Bagnato 2025 [69]	Excluded	AI performance evaluation (ChatGPT-4o/o1 accuracy on disorders-of-consciousness questions); no acceptance construct
Barbano 2024 [70]	Excluded	AI-assisted COVID-19 chest X-ray detection; performance study without acceptance component
Barbuiani 2026 [71]	Excluded	Italian scoping review on nurses' role in deprescribing without AI component; outside the operational concept of AI acceptance
Bevilacqua 2025 [72]	Excluded	Italian PRISMA-ScR-compliant scoping review on Human-Centred AI; review without primary empirical data on attitudes, acceptance, or perceptions of AI in healthcare
Bignami 2025 [73]	Excluded	Comment on Green AI and environmental impact in Italian hospitals without empirical acceptance assessment
Blagec 2018 [74]	Excluded	Conference paper. European clinical decision support implementation paper without empirical assessment of attitudes, acceptance, or perceptions; technical infrastructure description without an Italian sub-sample of attitudinal data
Bragazzi 2023 [75]	Excluded	International PRISMA-ScR-compliant scoping review on generative conversational AI in LGBTQ mental health; review without primary empirical data on attitudes, acceptance, or perceptions of AI in healthcare
Buongiorno 2025 [76]	Excluded	Italian scoping review on cognitive biases in forensic psychiatry; AI peripheral as bias-mitigation tool only
Cangelosi 2025 [77]	Excluded	Italian PRISMA-ScR-compliant scoping review on barriers and facilitators of AI implementation in diabetes management from a healthcare professional perspective; review without primary empirical data on attitudes, acceptance, or perceptions of AI in healthcare
Carriero 2026 [34]	Included	SIRM Young Committee national survey of Italian radiology residents and young radiologists (n = 204) with dedicated AI section
Carulli 2025 [78]	Excluded	Consensus study among Italian orthopaedic surgeons using an AI tool; AI acceptance only a secondary component, primary focus orthopaedic
Casà 2021 [35]	Included	National survey of Italian young physicians (SIGM) on digital competencies; AI-readiness baseline data (n = 362)
Cascella 2024 [36]	Included	Italian cross-sectional study on medical attitudes towards AI use in fibromyalgia management at the Annual Thinking Lab on Fibromyalgia Syndrome (ATLAS 2024) congress in Gubbio (n = 26)
Catelli 2023 [79]	Excluded	Italian Twitter sentiment analysis on COVID-19 vaccines; NLP as analytic tool, not study object

First author, year [Reference]	Decision	Reason / Note on inclusion or exclusion
Cavallucci 2026 [26]	Included	Cross-sectional survey of oncology patients at IRCCS IRST Dino Amadori on AI attitudes and data protection (n = 117)
Cavasin 2026 [54]	Included	Italian validation of the AIDUA-IT scale (University of Padova; n = 140); eight-factor confirmatory model with CFI = 0.984
Cè 2024 [37]	Included	Survey among Italian radiologists in Lombardy (n = 232) on AI integration with satisfaction, innovation readiness, AI literacy and optimism measures
Chamouni 2025 [80]	Excluded	International PRISMA-ScR-compliant scoping review on ethical and legal concerns in AI for lung cancer care; review without primary empirical data on attitudes, acceptance, or perceptions of AI in healthcare
Cicero 2025 [27]	Included	Italian validation of the General Attitudes towards Artificial Intelligence Scale (GAAIS-IT); two-study confirmatory factor analysis (n = 236)
Ciet 2022 [81]	Excluded	State-of-the-art review on cystic fibrosis lung imaging; AI peripheral, without acceptance component
Cingolani 2022 [82]	Excluded	Perspective article on AI and digitalisation in Italian home care without empirical acceptance assessment
Ciulli 2026 [38]	Included	Multicentre cross-sectional survey of Tuscan emergency-department triage nurses (n = 84) on AI perceptions
Cofini 2025 [56]	Included	Development and validation of the Italian I-KAPCAM-AI-Q questionnaire for healthcare provider AI awareness; n = 203 pilot
Cofini 2026 [53]	Included	Italian national application of the I-KAPCAM-AI-Q on physicians' knowledge, attitudes and concordance with AI-generated diagnoses (n = 587)
Coppola 2021 [39]	Included	Italian radiologists' nationwide survey on AI expectations and opinions (SIRM membership; n = 1,032)
Cosma 2025 [83]	Excluded	Italian PRISMA-ScR-compliant scoping review on chatbots and large language models for vaccine literacy; review without primary empirical data on attitudes, acceptance, or perceptions of AI in healthcare
Costantino 2022 [84]	Excluded	Conference paper. Italian ECCO conference abstract on telemedicine acceptance in IBD patients (AMICI Onlus); no AI acceptance construct, focus on telemedicine and televisits without AI component
D'Ambrosio 2024 [85]	Excluded	PRECISE study protocol without reported results at the time of screening
D'Amone 2025 [86]	Excluded	Italian online tendinopathy content analysis using machine learning data mining as analytic tool
Dagliati 2018 [87]	Excluded	Rule-based clinical decision support without machine learning component; outside the operational AI definition (machine learning, deep learning, large language models)

First author, year [Reference]	Decision	Reason / Note on inclusion or exclusion
Danieli 2021 [40]	Included	Italian conversational AI mental health agent feasibility with clinician acceptance evaluation (n = 21)
De Angelis 2025 [88]	Excluded	Commentary on the Learning Health System framework for AI integration in Italy without empirical assessment
De Batlle 2023 [89]	Excluded	Tutorial-format methodological implementation guide for the GATEKEEPER multinational eHealth piloting; no empirical acceptance assessment
De Marco 2025 [90]	Excluded	Conference paper. Italian WORDSforPAIN project conference abstract on test-retest reliability of a qualitative pain assessment model; technical reliability evaluation of an AI-based instrument without empirical assessment of attitudes, acceptance, or perceptions
Dellafiore 2026 [41]	Included	Reflexive thematic analysis with Italian expert qualitative researchers on AI in qualitative inquiry (n = 14)
Deodato 2025 [42]	Included	Italian national survey of physiotherapists (AIFI; n = 415) on knowledge, use and perceptions of AI chatbots; corresponding author at Hospital of Merano (SABES-ASDAA)
Di Nicola 2025 [91]	Excluded	Italian multicentre real-world study on patient experience with intranasal esketamine; no AI component
Doglietto 2020 [92]	Excluded	COVID-19 anxiety study in neurosurgical patients; no AI component
Dondi 2024 [93]	Excluded	Italian short communication on Real World Evidence in pre-marketing drug phase; AI mentioned only prospectively
Fanelli 2026 [94]	Excluded	Strategic implementation roadmap for the regional digital pathology network in Tuscany without empirical acceptance assessment
Fietta 2025 [95]	Excluded	Pilot feasibility study protocol of a chatbot-based stress management intervention; no reported results
Fiorini 2021 [57]	Included	Multinational ACCRA project (Italy and the Netherlands) qualitative needs and attitudes study on assistive robotics in older adults and carers; mixed populations (20 older people + 34 caregivers)
Forte 2025 [43]	Included	Cross-sectional study among Italian rheumatologists and patients with psoriatic arthritis (n = 76 patients, n = 12 specialists); blinded comparison of ChatGPT versus expert responses with patient preference as primary endpoint
Garagiola 2021 [96]	Excluded	Conference paper. Italian conference abstract on T-CUBE telemedicine platform acceptability; AI mentioned as background context only, the studied tool is a telemonitoring platform without AI-decision component. The corresponding full-length publication (Foglia et al., BMC Health Serv Res, 2024) describes the same T-CUBE study and would equally not satisfy

First author, year [Reference]	Decision	Reason / Note on inclusion or exclusion
		the AI-acceptance inclusion criterion; the conference record and its full-length counterpart are accordingly treated as a single excluded study
Gastaldi 2025 [97]	Excluded	PRISMA-ScR-compliant scoping review on AI in infection prevention and control; review without primary empirical data on attitudes, acceptance, or perceptions of AI in healthcare
Giannella 2024 [27]	Included	IRCCS Santa Lucia Rome cross-sectional study (n = 1,454) on biobanking, data use and privacy attitudes among healthy volunteers and neurological patients
Giansanti 2025 [44]	Included	Italian survey (Istituto Superiore di Sanità) of n = 150 digital cytology professionals on AI familiarity, perceived benefits and adoption barriers
Giordani 2026 [98]	Excluded	Brief note on the AULSS6 Padova AI implementation strategy; conceptual paper without empirical acceptance assessment
Giubilato 2025 [99]	Excluded	ANMCO position paper on cardiovascular chronicity management; AI mentioned programmatically without empirical assessment
Godoy Junior 2022 [100]	Excluded	Conference paper. International conference abstract on patients' and neurologists' preferences for remote monitoring and AI in Parkinson's disease; multinational sample without identifiable Italian sub-sample for the AI-acceptance component
Hesso 2023 [58]	Included	Multinational mixed-methods study (six European countries, INCISIVE-EU H2020) on healthcare professionals' AI perceptions in cancer care; substantial Italian sub-sample (26/95 = 27.4 % survey respondents; 7/27 = 25.9 % interview participants)
Hesso 2024 [59]	Included	Sister publication to Hesso 2023 in INCISIVE-EU project; e-mail interviews with oncology specialists from seven European countries (n = 10) including Italian sub-sample (n = 2)
Iannone 2023 [101]	Excluded	Italian narrative review on AI in autism care; review without primary empirical data on attitudes, acceptance, or perceptions of AI in healthcare
Ihaddouchen 2025 [102]	Excluded	Conference paper. European multicentre conference abstract on ICU physicians' AI acceptance; full text not retrievable through supplementary searches (PRISMA-ScR Item 16); without retrievable full text the substantial Italian sub-sample requirement could not be verified
Iosca 2025 [103]	Excluded	Conference paper. Italian ESTRO 2025 conference abstract on radiotherapy technologists' AI attitudes in two Italian regions; no full publication identified, considered as conference-only record outside the operational definition of full empirical publication
Kahlawi 2025 [104]	Excluded	Cross-cultural Twitter text-mining COVID-19 study; NLP as analytic tool, not study object
Krakowski 2026 [105]	Excluded	United Kingdom causal-machine-learning study for first-line antipsychotic recommendations; algorithm performance without acceptance assessment

First author, year [Reference]	Decision	Reason / Note on inclusion or exclusion
La Regina 2025 [28]	Included	Nationally representative Italian DOXA panel survey (n = 1,200) on patient safety culture including AI sub-component
Laborie 2023 [106]	Excluded	ESPR AI taskforce position paper without empirical study design
Lusi 2022 [107]	Excluded	Conference paper. Italian conference abstract on AI in head and neck tumour multidisciplinary management; no identifiable full publication retrieved through supplementary searches
Magni 2021 [108]	Excluded	Conference paper. Italian conference abstract on web-listening NLP analysis of cardiovascular discussions during COVID-19; NLP used as analytic tool without AI acceptance construct
Mahlknecht 2023 [29]	Included	Feasibility study on AI symptom checker in South Tyrolean primary care with patient and physician acceptance assessment (Claudiana, Bolzano; 10 GPs + 116 patients analysed)
Maida 2025 [45]	Included	Italian national survey of gastroenterologists on AI perceptions in endoscopy (n = 150)
Maina 2025 [109]	Excluded	Conference paper. Italian survey (n = 73 clinicians) on perceptions of AI in medical writing (ISMPP European Meeting 2025); AI application restricted to manuscript and abstract preparation rather than clinical, diagnostic, therapeutic, or patient-facing healthcare delivery, and therefore outside the operational definition of AI in healthcare adopted for this scoping review.
Maines 2020 [110]	Excluded	Conference paper. Italian conference abstract on TreC-cardio tele-cardiology system in Trentino; technical and organisational description without empirical assessment of attitudes, acceptance, or perceptions of AI
Maran 2025 [111]	Excluded	Scoping review on therapeutic radiographers' research trends; AI peripheral
Martin 2024 [112]	Excluded	Screen4Care study protocol on parental preferences for genetic newborn screening; AI as background technology only
Mizzi 2024 [113]	Excluded	Multinational qualitative study on supplementary breast cancer screening implementation; AI only one of several solution options
Monaco 2019 [114]	Excluded	Brain–computer interface treatment study without acceptance or attitudinal component
Monaco 2024 [115]	Excluded	AI platform description for eating disorder care; no empirical acceptance assessment
Montagna 2020 [116]	Excluded	Vaccination policy adherence survey without AI or acceptance component
Nabata 2025 [117]	Excluded	Canadian study on human distinguishability of ChatGPT-generated surgical content; outside Italian or comparator scope

First author, year [Reference]	Decision	Reason / Note on inclusion or exclusion
Osnat 2025 [118]	Excluded	Global PRISMA-ScR-compliant scoping review on patient AI perspectives in healthcare; review without primary empirical data on attitudes, acceptance, or perceptions of AI in healthcare
Oznur 2024 [119]	Excluded	Conference paper. International conference abstract on bibliometric analysis of AI in pain research; bibliometric study without empirical assessment of attitudes, acceptance, or perceptions
Park 2023 [120]	Excluded	Conference paper. South Korean conference abstract on decentralised clinical trial elements feasibility; no Italian context, no Italian population, and no AI acceptance construct
Perrella 2026 [25]	Included	Italian cross-sectional survey of clinicians on the optimism–knowledge gap (n = 362) within the Garante della Privacy regulatory framework
Pesapane 2023 [30]	Included	Prospective survey at IEO Milan (n = 800 women) on AI knowledge, attitudes and accountability perceptions in mammography screening
Pesapane 2026 [32]	Included	Randomised experimental survey on AI disclosure in mammography reports among 600 women in two academic centres in Milan (Eur Radiol)
Pesapane 2026 [31]	Included	Italian Cancer Referral Centre patient survey on AI perceptions in medicine and radiology (n = 240); Esmaeilzadeh-model adaptation (JCO Clin Cancer Inform)
Piazza 2021 [121]	Excluded	Conference paper. Italian conference abstract on prostate-cancer online forum interactions; descriptive analysis without AI component or acceptance construct
Piras 2025 [46]	Included	Italian young radiation oncologists yAIRO survey on ChatGPT use (n = 76)
Ramacciati 2023 [122]	Excluded	Nursing diagnosis terminology validation through retrospective documentation; no AI component
Ravaldi 2023 [123]	Excluded	Perinatal emotion analysis during COVID-19 using NLP/ML as method, not study object
Rizzo 2026 [47]	Included	First AMCLI/GLAIMAL national survey on AI and machine learning adoption in Italian clinical microbiology laboratories (n = 163)
Rossero 2026 [48]	Included	Qualitative study with 24 Italian surgeons and radiologists on trust, risk and professional boundaries in robotic and AI-enhanced care
Runge 2025 [124]	Excluded	Editorial on magnetic resonance contrast media; AI peripheral
Seregni 2022 [125]	Excluded	SIAMOC conference abstract on virtual coaching pilot; no acceptance assessment of the system
Shahrabani 2024 [126]	Excluded	Conference paper. International conference abstract on Anki spaced-repetition flashcards in medical education; software description without AI acceptance construct, no Italian context
Shalom 2022 [127]	Excluded	Israeli–Dutch implementation study without Italian context or acceptance assessment

First author, year [Reference]	Decision	Reason / Note on inclusion or exclusion
Ting 2025 [128]	Excluded	Conference paper. International conference abstract on AI integration in health technology assessments; HTA-policy review without primary empirical data on attitudes, acceptance, or perceptions of AI in healthcare in clinical settings
Torricelli 2025 [49]	Included	Northern Italian hospital physicians' survey on AI acceptance, fears and expectations (n = 176)
Tortella 2025 [50]	Included	Italian national cross-sectional survey of physiotherapy students on AI chatbot knowledge and use (n = 589); funding from Autonomous Province of Bozen/Bolzano
Tozzi 2023 [51]	Included	Italian national survey of paediatricians (Bambino Gesù IRCCS; n = 1,540) on educational priorities for emerging technologies including AI
Van Cauwenberge 2022 [129]	Excluded	Belgian qualitative interview study (n = 24, all participants from Ghent University Hospital); no Italian context, no Italian population, and no Italian sub-sample
Varrassi 2026 [130]	Excluded	Scoping review on cancer pain pathophysiology and management; AI peripheral as one therapeutic component among several
Vignapiano 2024 [131]	Excluded	Sister study protocol/platform description for autism spectrum disorder care; no reported results
Visci 2025 [132]	Excluded	Retrospective four-year analysis of forensic consultations at Policlinico Bari; AI mentioned only peripherally in discussion
Vozzi 2026 [52]	Included	National web-based survey of Italian neurology residents on AI training (n = 173)
Zavattaro 2026 [133]	Excluded	Tutorial on the Trust Analysis Canvas in Swiss academic settings; no Italian context or empirical acceptance assessment

Records are listed in alphabetical order by first author. Both full-length journal articles and conference papers retrieved through the database searches are included. Eligibility for inclusion required (i) primary empirical data on attitudes, acceptance, or perceptions of AI in healthcare and (ii) an Italian context, an Italian population, or a substantial Italian sub-sample. Reviews and other secondary syntheses without primary empirical data, conference abstracts without retrievable full publications, and studies without Italian context or sub-sample were therefore excluded. Conference papers were treated under the JBI guidance for scoping reviews (Peters et al., 2024) and PRISMA-ScR Items 9 and 13 (Tricco et al., 2018). Records flagged in the Reason column as 'Conference paper' were assessed for eligibility on their merits when no corresponding full-length publication could be identified. Where a corresponding full-length journal publication of the same study was retrieved through supplementary searches (Garagiola 2021 / Foglia 2024 on the T-CUBE telemedicine platform), the conference record and its full-length counterpart were treated as a single record and assessed jointly. One conference record (Ihaddouchen 2025) could not be retrieved in full text and is documented per PRISMA-ScR Item 16. Abbreviations: AI, artificial intelligence; AIDUA, Artificially Intelligent Device Use Acceptance; AIFI, Associazione Italiana di Fisioterapia; AMCLI, Associazione Microbiologi Clinici Italiani; AMICI, Associazione Nazionale per le

Malattie Infiammatorie Croniche dell'Intestino; ANMCO, Associazione Nazionale Medici Cardiologi Ospedalieri; ATLAS, Annual Thinking Lab on Fibromyalgia Syndrome; AULSS, Azienda Unità Locale Socio Sanitaria; CFI, comparative fit index; ECCO, European Crohn's and Colitis Organisation; ESPR, European Society of Paediatric Radiology; ESTRO, European Society for Radiotherapy and Oncology; GAAIS, General Attitudes towards Artificial Intelligence Scale; GLAIMAL, Gruppo di Lavoro AI/ML in Microbiologia; GP, general practitioner; HCP, healthcare professional; HTA, health technology assessment; ICU, intensive care unit; IEO, European Institute of Oncology; I-KAPCAM-AI-Q, Italian Knowledge, Attitudes, Practices and Clinical Application of Medical AI Questionnaire; IRCCS, Istituto di Ricovero e Cura a Carattere Scientifico; IRST, Istituto Romagnolo per lo Studio dei Tumori; JBI, Joanna Briggs Institute; LLM, large language model; ML, machine learning; NLP, natural language processing; PRISMA-ScR, Preferred Reporting Items for Systematic reviews and Meta-Analyses extension for Scoping Reviews; SABES-ASDAA, Azienda Sanitaria dell'Alto Adige; SIAMOC, Società Italiana di Analisi del Movimento in Clinica; SIGENP, Società Italiana di Gastroenterologia, Epatologia e Nutrizione Pediatrica; SIIAM, Società Italiana per l'Intelligenza Artificiale in Medicina; SIRM, Società Italiana di Radiologia Medica e Interventistica; yAIRO, young Italian Association of Radiation and Clinical Oncology.

References

25. Perrella, A.; di Micco, P.; Trama, U.; di Silverio, P.; Maffettone, A.; Piccinocchi, G.; Bernardi, F.F. Assessing the “Optimism-Knowledge Gap”: An Exploratory Study of AI Awareness, Application, and Educational Needs Among a Sample of Italian Clinicians. Healthcare 2026, 14, 847. https://doi.org/10.3390/healthcare14070847.
26. Cavallucci, M.; Andalò, A.; Danesi, V.; Gentili, N.; Massa, I.; Scarpi, E.; Restuccia, M.C.; Vespignani, R.; Conficconi, A.; Palleschi, M.; et al. Survey on Cancer Patients’ Attitudes towards AI and Data Protection: A Cross-Sectional Study from an Italian Cancer Center. Int. J. Med. Inform. 2026, 209, 106237. https://doi.org/10.1016/j.ijmedinf.2025.106237.
27. Giannella, E.; Bauça, J.M.; Di Santo, S.G.; Brunelli, S.; Costa, E.; Di Fonzo, S.; Fusco, F.R.; Perre, A.; Pisani, V.; Presicce, G.; et al. Biobanking, Digital Health and Privacy: The Choices of 1410 Volunteers and Neurological Patients Regarding Limitations on Use of Data and Biological Samples, Return of Results and Sharing. BMC Med. Ethics 2024, 25, 100. https://doi.org/10.1186/s12910-024-01102-3.
28. La Regina, M.; Parretti, C.; Bernardini, D.; Oneto, N.; Torricelli, G.; Federici, L.; Tartaglia, R. Assessment of Patient Safety Culture Among Citizens: A Survey Study. J. Patient Saf. 2025, 21, 530–536. https://doi.org/10.1097/PTS.0000000000001391.
29. Mahlknecht, A.; Engl, A.; Piccoliori, G.; Wiedermann, C.J. Supporting Primary Care through Symptom Checking Artificial Intelligence: A Study of Patient and Physician Attitudes in Italian General Practice. BMC Prim. Care 2023, 24, 174. https://doi.org/10.1186/s12875-023-02143-0.
30. Pesapane, F.; Rotili, A.; Valconi, E.; Agazzi, G.M.; Montesano, M.; Penco, S.; Nicosia, L.; Bozzini, A.; Meneghetti, L.; Latronico, A.; et al. Women’s Perceptions and Attitudes to the Use of AI in Breast Cancer Screening: A Survey in a Cancer Referral Centre. Br. J. Radiol. 2023, 96, 20220569. https://doi.org/10.1259/bjr.20220569.
31. Pesapane, F.; Giambersio, E.; Rotili, A.; Grasso, R.; Gaeta, A.; Battaglia, O.; Conti, L.; Pizzoli, S.F.M.; Raimondi, S.; Gandini, S.; et al. Public Perspectives on Artificial Intelligence in Medicine and Radiology: Insights From a Survey in an Italian Cancer Referral Center. JCO Clin. Cancer Inform. 2026, 10, e2500210. https://doi.org/10.1200/CCI-25-00210.
32. Pesapane, F.; Depretto, C.; Rotili, A.; Penco, S.; Monzani, D.; Grasso, R.; Nicosia, L.; Mallardi, C.; D’Amelio, L.; Carriero, S.; et al. Should AI Results Be Disclosed in Mammography Reports? A Randomised Survey Study of Patient Responses to Concordant and Discordant Interpretations. Eur. Radiol. 2026. https://doi.org/10.1007/s00330-026-12405-x.
33. Ancona, S.; Malerba, F.; Medina, F.; Gandullia, P.; Chiaro, A.; Renzo, S.; De Angelis, P.; Romano, C.; Malamisura, M.; Auricchio, R.; et al. Adherence to Guidelines, Pitfalls, and Emerging Technologies in Endoscopic Practice for Celiac Disease: A Nationwide SIGENP Survey. Eur. J. Pediatr. 2026, 185, 173.

34. Carriero, S.; Lorusso, G.; Ce', M.; Chiti, G.; Bruno, A.; Grassi, F.; Magnaldi, S.; Borrè, A.; Gandolfo, N.; Giovagnoni, A.; et al. The Real World of Italian New-Generation Radiologists: Challenges and Career Expectations. Radiol. Med. 2026, 131, 656–668. https://doi.org/10.1007/s11547-025-02127-3.
35. Casà, C.; Marotta, C.; Di Pumpo, M.; Cozzolino, A.; D'Aviero, A.; Frisicale, E.M.; Silenzi, A.; Gabbrielli, F.; Bertinato, L.; Brusaferro, S. COVID-19 and Digital Competencies among Young Physicians: Are We (Really) Ready for the New Era? A National Survey of the Italian Young Medical Doctors Association. Ann. Dell'istituto Super. Sanità 2021, 57, 1–6. https://doi.org/10.4415/ANN_21_01_01.
36. Cascella, M.; Guerra, C.; De Feo, R.; Cerrone, V.; Farah, S.; Sarzi-Puttini, P.; Salaffi, F. Cross-Sectional Study on Medical Attitude Towards Artificial Intelligence Use in Fibromyalgia: Insights From the Annual Thinking Lab on Fibromyalgia Syndrome (ATLAS 2024). Transl. Med. UniSa 2024, 26, 153–163. https://doi.org/10.37825/2239-9747.1066.
37. Cè, M.; Ibba, S.; Cellina, M.; Tancredi, C.; Fantesini, A.; Fazzini, D.; Fortunati, A.; Perazzo, C.; Presta, R.; Montanari, R.; et al. Radiologists' Perceptions on AI Integration: An in-Depth Survey Study. Eur. J. Radiol. 2024, 177, 111590. https://doi.org/10.1016/j.ejrad.2024.111590.
38. Ciulli, A.; Ceccanti, L.; Gheri, F. Triage Nurses' Perceptions of Artificial Intelligence: A Cross-Sectional Study. J. Emerg. Nurs. 2026, in press. https://doi.org/10.1016/j.jen.2026.02.001.
39. Coppola, F.; Faggioni, L.; Regge, D.; Giovagnoni, A.; Golfieri, R.; Bibbolino, C.; Miele, V.; Neri, E.; Grassi, R. Artificial Intelligence: Radiologists' Expectations and Opinions Gleaned from a Nationwide Online Survey. Radiol. Med. 2021, 126, 63–71. https://doi.org/10.1007/s11547-020-01205-y.
40. Danieli, M.; Ciulli, T.; Mousavi, S.M.; Riccardi, G. A Conversational Artificial Intelligence Agent for a Mental Health Care App: Evaluation Study of Its Participatory Design. JMIR Form. Res. 2021, 5, e30053. https://doi.org/10.2196/30053.
41. Dellafiore, F.; Saba, A.; Collaro, C.; Artioli, G. Artificial Intelligence in Qualitative Research: Insights From Experts via Reflexive Thematic Analysis. Qual. Health Res. 2026, 36, 145–165. https://doi.org/10.1177/10497323251389800.
42. Deodato, M.; Sabot, R.; Galmonte, A.; Palese, A.; Castellini, G.; Turolla, A.; Pillastrini, P.; Cook, C.; Rodeghiero, L.; Gianola, S.; et al. Knowledge, Use and Perceptions of Artificial Intelligence Chatbots among Italian Physiotherapists: An Online Cross-Sectional Survey. Front. Digit. Health 2025, 7, 1671521. https://doi.org/10.3389/fdgth.2025.1671521.
43. Forte, G.; Mauro, D.; Raimondi, M.; Pantano, I.; Gandolfo, S.; Cauli, A.; Guggino, G.; Lubrano, E.; Guiducci, S.; Chimenti, M.S.; et al. ChatGPT vs Rheumatologists: Cross-Sectional Study on Accuracy and Patient Perception of AI-Generated Information for Psoriatic Arthritis. Ann. Rheum. Dis. 2025, in press.
44. Giansanti, D.; Carico, E.; Lastrucci, A.; Giarnieri, E. Surveying the Digital Cytology Workflow in Italy: An Initial Report on AI Integration Across Key Professional Roles. Healthcare 2025, 13, 903. https://doi.org/10.3390/healthcare13080903.
45. Maida, M.; Sferrazza, S.; Calabrese, G.; Marasco, G.; Vitello, A.; Furnari, M.; Boskoski, I.; Sinagra, E.; Facciorusso, A. Perceptions of Artificial Intelligence Among Gastroenterologists in Italy: A National Survey. Cancers 2025, 17, 1353.
46. Piras, A.; Mastroleo, F.; Colciago, R.R.; Morelli, I.; D'Aviero, A.; Longo, S.; Grassi, R.; Iorio, G.C.; De Felice, F.; Boldrini, L.; et al. How Italian Radiation Oncologists Use ChatGPT: A Survey by the Young Group of the Italian Association of Radiotherapy and Clinical Oncology (yAIRO). Radiol. Med. 2025, 130, 453–462.
47. Rizzo, A.; Mensa, E.; Squarzon, L.; Clerici, P.; Lucis, R. Artificial Intelligence in Clinical Microbiology: Results from the First National Survey by the Italian Association of Clinical Microbiologists. Eur. J. Clin. Microbiol. Infect. Dis. 2026, 45, 197–206. https://doi.org/10.1007/s10096-025-05317-z.
48. Rossero, E.; Lombi, L. Recalibrating Expectations in Robotic and AI-Enhanced Care: Trust, Risk, and Professional Boundaries Renegotiation. Health Risk Soc. 2026, 28, 172–191. https://doi.org/10.1080/13698575.2026.2646879.
49. Torricelli, P.; Torricelli, C.; Bertelli, B.; Sandi, M.; Pecchi, A. Physician Perceptions of Artificial Intelligence in Northern Italy Healthcare: A Survey of Fears and Expectations. Front. Artif. Intell. 2025, 8, 1624789. https://doi.org/10.3389/frai.2025.1624789.
50. Tortella, F.; Palese, A.; Turolla, A.; Castellini, G.; Pillastrini, P.; Landuzzi, M.G.; Cook, C.; Galeoto, G.; Giovannico, G.; Rodeghiero, L.; et al. Knowledge and Use, Perceptions of Benefits and Limitations of Artificial Intelligence Chatbots among Italian Physiotherapy Students: A Cross-Sectional National Study. BMC Med. Educ. 2025, 25, 572. https://doi.org/10.1186/s12909-025-07176-w.

51. Tozzi, A.E.; Gesualdo, F.; Pandolfi, E.; Ferro, D.; Cinelli, G.; Bozzola, E.; Aversa, T.; Di Mauro, A.; Mameli, C.; Croci, I. Prioritizing Educational Initiatives on Emerging Technologies for Italian Pediatricians: Bibliometric Review and a Survey. Ital. J. Pediatr. 2023, 49, 112. https://doi.org/10.1186/s13052-023-01512-w.
52. Vozzi, C.; Sibilla, M.; Sandri, D.; Marinato, V.; Micolonghi, G.; Oliveri, S.; Filippi, M.; Marceglia, S.; Priori, A. Education Research: Bridging the Artificial Intelligence Training Gap: Evidence from a National Survey of Italian Neurology Residents. Neurol. Educ. 2026, 5, e200289. https://doi.org/10.1212/NE9.0000000000200289.
53. Cofini, V.; Muselli, M.; Piccardi, L.; Benvenuti, E.; Di Pangrazio, G.; Mancinelli, M.; Cimino, E.; Palermo, P.; Petrucci, E.; Picchi, G.; et al. Medical Clinical Minds Meet Artificial Intelligence: Italian Physicians' Knowledge, Attitudes, and Concordance between Italian Physicians and AI-Generated Diagnoses. A National Cross-Sectional Study. Front. Digit. Health 2026, 8, 1787117. https://doi.org/10.3389/fdgth.2026.1787117.
54. Cavasin, G.; Ocagli, H.; Gregori, D. Preliminary Validation of the Italian Version of the Artificially Intelligent Device Use Acceptance (AIDUA-IT) Scale: Cross-Cultural Adaptation and Psychometric Evaluation. J. Clin. Med. 2026, 15, 1578. https://doi.org/10.3390/jcm15041578.
55. Cicero, L.; Russo, A.; Di Stefano, G.; Zammitti, A. The General Attitudes towards Artificial Intelligence Scale (GAAIS): Validation and Psychometric Properties Analysis in the Italian Context. BMC Psychol. 2025, 13, 641.
56. Cofini, V.; Piccardi, L.; Benvenuti, E.; Di Pangrazio, G.; Cimino, E.; Mancinelli, M.; Muselli, M.; Petrucci, E.; Picchi, G.; Palermo, P.; et al. The I-KAPCAM-AI-Q: A Novel Instrument for Evaluating Health Care Providers' AI Awareness in Italy. Front. Public Health 2025, 13, 1655659. https://doi.org/10.3389/fpubh.2025.1655659.
57. Fiorini, L.; De Mul, M.; Fabbricotti, I.; Limosani, R.; Vitanza, A.; D'Onofrio, G.; Tsui, M.; Sancarlo, D.; Giuliani, F.; Greco, A.; et al. Assistive Robots to Improve the Independent Living of Older Persons: Results from a Needs Study. Disabil. Rehabil. Assist. Technol. 2021, 16, 92–102. https://doi.org/10.1080/17483107.2019.1642392.
58. Hesso, I.; Kayyali, R.; Dolton, D.-R.; Joo, K.; Zacharias, L.; Charalambous, A.; Lavdaniti, M.; Stalika, E.; Ajami, T.; Acampa, W.; et al. Cancer Care at the Time of the Fourth Industrial Revolution: An Insight to Healthcare Professionals' Perspectives on Cancer Care and Artificial Intelligence. Radiat. Oncol. 2023, 18, 167. https://doi.org/10.1186/s13014-023-02351-z.
59. Hesso, I.; Kayyali, R.; Zacharias, L.; Charalambous, A.; Lavdaniti, M.; Stalika, E.; Ajami, T.; Acampa, W.; Boban, J.; Gebara, S.N. Cancer Care Pathways across Seven Countries in Europe: What Are the Current Obstacles? And How Can Artificial Intelligence Help? J. Cancer Policy 2024, 39, 100457. https://doi.org/10.1016/j.jcpo.2023.100457.
60. Negash, S.; Gundlack, J.; Buch, C.; Apfelbacher, T.; Schildmann, J.; Frese, T.; Christoph, J.; Mikolajczyk, R. Physicians' Attitudes and Acceptance towards Artificial Intelligence in Medical Care: A Qualitative Study in Germany. Front. Digit. Health 2025, 7, 1616827. https://doi.org/10.3389/fdgth.2025.1616827.
61. Statistisches Landesinstitut der Autonomen Provinz Bozen-Südtirol (ASTAT). Nutzung von KI Und Meinungen Zur KI in Der Gesundheitsversorgung–Februar 2026/Uso Dell'IA e Opinioni Sull'IA Nell'assistenza Sanitaria–Febbraio 2026; Astat Info; Statistisches Landesinstitut der Autonomen Provinz Bozen-Südtirol (ASTAT): Bozen/Bolzano, Italy, 2026.
62. Álvarez Sánchez-Bayuela, D.; Fernández Martín, J.; Tiberi, G.; Ghavami, N.; Giovanetti González, R.; Cruz Hernánez, L.M.; Aguilar Angulo, P.M.; Martínez Gómez, A.D.; Rodríguez Sánchez, A.; Bigotti, A. Microwave Imaging for Breast Cancer Screening: Protocol for an Open, Multicentric, Interventional, Prospective, Non-Randomised Clinical Investigation to Evaluate Cancer Detection Capabilities of MammoWave System on an Asymptomatic Population across Multiple European Countries. BMJ Open 2024, 14, e088431.
63. Amabili, G.; Maranesi, E.; Margaritini, A.; Bonfigli, A.R.; Felici, E.; Barbarossa, F.; Benadduci, M.; Gosetto, L.; Guebey, J.; Grimstad, T.; et al. Managing Cognitive Decline Through a Social Robot-Based Intervention: Protocol for the engAGE Proof of Concept and Randomized Controlled Trial. JMIR Res. Protoc. 2025, 14, e67601. https://doi.org/10.2196/67601.
64. Antón-Rodríguez, C.; Medina, M.; Rodríguez, P.; Barahona, Á.; Álvarez-Montero, S. Accompaniment in the Healthcare Sector: A Systematic Review and Concept Analysis. Front. Med. 2026, 13, 1724133. https://doi.org/10.3389/fmed.2026.1724133.
65. Arcà, E.; Ames, J.T.; Avissar, J.; Santpurkar, N.; Fox, G.E. HPR79 Unlocking AI's Potential in Pricing and Reimbursement: Insights Across Global Healthcare Archetypes. Value Health 2025, 28, S207.
66. Arzilli, G.; De Vita, E.; Pasquale, M.; Carloni, L.M.; Pellegrini, M.; Di Giacomo, M.; Esposito, E.; Porretta, A.D.; Rizzo, C. Innovative Techniques for Infection Control and Surveillance in Hospital Settings and Long-Term Care Facilities: A Scoping Review. Antibiotics 2024, 13, 77.

67. Baglivo, F.; Diedenhofen, G.; De Angelis, L.; Pivetta, A.; Causio, F.A.; D'Ambrosio, A.; Sacchi, F.A.; Di Pumpo, M.; Belpiede, A.; Ghisalberti, G.; et al. Why Tomorrow's Public Health Needs to Be Digital: Artificial Intelligence and Automation for a Sustainable Italian National Health Service. Recenti Prog. Med. 2025, 116, 551–555. https://doi.org/10.1701/4573.45775.
68. Baglivo, F.; Ferro, D.; Diedenhofen, G. [Artificial intelligence for italian medicine: Navigating between innovation and practice]. Recenti Prog. Med. 2025, 116, 543–545. https://doi.org/10.1701/4573.45773.
69. Bagnato, S.; Boccagni, C.; Bonavita, J. Assessing the Accuracy of ChatGPT in Answering Questions About Prolonged Disorders of Consciousness. Brain Sci. 2025, 15, 392. https://doi.org/10.3390/brainsci15040392.
70. Barbano, C.A.; Berton, L.; Renzulli, R.; Tricarico, D.; Rampado, O.; Basile, D.; Busso, M.; Grosso, M.; Grangetto, M. Detection and Prioritization of COVID-19 Infected Patients from CXR Images: Analysis of AI-Assisted Diagnosis in Clinical Settings. Comput. Struct. Biotechnol. J. 2024, 24, 754–761. https://doi.org/10.1016/j.csbj.2024.11.045.
71. Barbuiani, G.; Terzoni, S.; Caruso, R.; Cillufo, S.; Pasina, L.; Lusignani, M. Nurses' Role in Deprescribing for Older Adults: A Scoping Review. Int. J. Nurs. Stud. 2026, 174, 105274.
72. Bevilacqua, R.; Bailoni, T.; Maranesi, E.; Amabili, G.; Barbarossa, F.; Ponzano, M.; Virgolesi, M.; Rea, T.; Illario, M.; Piras, E.M.; et al. Framing the Human-Centered Artificial Intelligence Concepts and Methods: Scoping Review. JMIR Hum. Factors 2025, 12, e67350. https://doi.org/10.2196/67350.
73. Bignami, E.; Darhour, L.J.; Bellini, V. Sustainable AI in Medicine: Navigating Innovation, Challenges, and Environmental Impact. Health Econ. Rev. 2025, 15, 110. https://doi.org/10.1186/s13561-025-00704-w.
74. Blagec, K.; Koopmann, R.; Crommentuijn-Van Rhenen, M.; Holsappel, I.; Van Der Wouden, C.; Konta, L.; Xu, H.; Steinberger, D.; Just, E.; Swen, J.J.; et al. Multi-Modal Decision Support to Enable Pharmacogenetics-Based Drug Dosing across Seven European Countries. Clin. Chem. Lab. Med. 2018, 56, eA108–eA109.
75. Bragazzi, N.L.; Crapanzano, A.; Converti, M.; Zerbetto, R.; Khamisy-Farah, R. The Impact of Generative Conversational Artificial Intelligence on the Lesbian, Gay, Bisexual, Transgender, and Queer Community: Scoping Review. J. Med. Internet Res. 2023, 25, e52091.
76. Buongiorno, L.; Mele, F.; Petroni, G.; Margari, A.; Carabellese, F.; Catanesi, R.; Mandarelli, G. Cognitive Biases in Forensic Psychiatry: A Scoping Review. Int. J. Law Psychiatry 2025, 101, 102083.
77. Cangelosi, G.; Conti, A.; Caggianelli, G.; Panella, M.; Petrelli, F.; Mancin, S.; Ratti, M.; Masini, A. Barriers and Facilitators to Artificial Intelligence Implementation in Diabetes Management from Healthcare Workers' Perspective: A Scoping Review. Medicina 2025, 61, 1403. https://doi.org/10.3390/medicina61081403.
78. Carulli, C.; Rossi, S.M.P.; Magistrelli, L.; Annibaldi, A.; Troncone, E. Can Artificial Intelligence Help Orthopaedic Surgeons in the Conservative Management of Knee Osteoarthritis? A Consensus Analysis. J. Clin. Med. 2025, 14, 690.
79. Catelli, R.; Pelosi, S.; Comito, C.; Pizzuti, C.; Esposito, M. Lexicon-Based Sentiment Analysis to Detect Opinions and Attitude towards COVID-19 Vaccines on Twitter in Italy. Comput. Biol. Med. 2023, 158, 106876. https://doi.org/10.1016/j.compbiomed.2023.106876.
80. Chamouni, G.; Lococo, F.; Sassorossi, C.; Atuhaire, N.; Ádány, R.; Varga, O. Ethical and Legal Concerns in Artificial Intelligence Applications for the Diagnosis and Treatment of Lung Cancer: A Scoping Review. Front. Public Health 2025, 13, 1663298. https://doi.org/10.3389/fpubh.2025.1663298.
81. Ciet, P.; Bertolo, S.; Ros, M.; Casciaro, R.; Cipolli, M.; Colagrande, S.; Costa, S.; Galici, V.; Gramegna, A.; Lanza, C.; et al. State-of-the-Art Review of Lung Imaging in Cystic Fibrosis with Recommendations for Pulmonologists and Radiologists from the "iMAging managEment of cySTic fibROsis" (MAESTRO) Consortium. Eur. Respir. Rev. 2022, 31, 210173. https://doi.org/10.1183/16000617.0173-2021.
82. Cingolani, M.; Scendoni, R.; Fedeli, P.; Cembrani, F. Artificial Intelligence and Digital Medicine for Integrated Home Care Services in Italy: Opportunities and Limits. Front. Public Health 2022, 10, 1095001. https://doi.org/10.3389/fpubh.2022.1095001.
83. Cosma, C.; Radi, A.; Cattano, R.; Zanobini, P.; Bonaccorsi, G.; Lorini, C.; Del Riccio, M. Exploring Chatbot Contributions to Enhancing Vaccine Literacy and Uptake: A Scoping Review of the Literature. Vaccine 2025, 44, 126559. https://doi.org/10.1016/j.vaccine.2024.126559.
84. Costantino, A.; Caprioli, F.; Vecchi, M.; Stocco, D.; Aloi, M.; Armuzzi, A.; Ficari, F.; Manguso, F.; Mocci, G.; Orlando, A.; et al. Telemedicine in IBD Patients: Results of a National Survey of the Italian IBD Patients' Association (AMICI Onlus). J. Crohn's Colitis 2022, 16, i481.
85. D'Ambrosio, F.; Harbo, M.; Contiero, D.; Bonfigli, A.R.; Cicconi, D.; Heuer, N.; Roos, A.; Fischer Pedersen, C.; Fabbietti, P.; Gagliardi, C. Preact to Lower the Risk of Falling by Customized Rehabilitation across Europe: The

Feasibility Study Protocol of the PRECISE Project in Italy. Front. Public Health 2024, 12, 1293621. https://doi.org/10.3389/fpubh.2024.1293621.
86. D'Amone, F.; Rio, E.; Esposto, M.; Ganderton, C.; Rossettini, G.; Cioeta, M. What Does Dr Google Tell Us about Tendinopathies? An Italian Analysis of Online Content. Pain. Manag. 2025, 15, 985–995. https://doi.org/10.1080/17581869.2025.2571389.
87. Dagliati, A.; Sacchi, L.; Tibollo, V.; Cogni, G.; Teliti, M.; Martinez-Millana, A.; Traver, V.; Segagni, D.; Posada, J.; Ottaviano, M.; et al. A Dashboard-Based System for Supporting Diabetes Care. J. Am. Med. Inform. Assoc. 2018, 25, 538–547.
88. De Angelis, L.; Pivetta, A.; Baglivo, F.; Cappellini, L.A.; Sacchi, F.A.; Di Pumpo, M.; Mercier, M.; Diedenhofen, G.; Di Bartolomeo, M.; Causio, F.A.; et al. Towards Learning Healthcare Systems in Italy: Opportunities and Challenges of AI at Point-of-Care. Recenti Prog. Med. 2025, 116, 556–560. https://doi.org/10.1701/4573.45776.
89. De Batlle, J.; Benítez, I.D.; Moncusí-Moix, A.; Androutsos, O.; Angles Barbastro, R.; Antonini, A.; Arana, E.; Cabrera-Umpierrez, M.F.; Cea, G.; Dafoulas, G.E.; et al. GATEKEEPER's Strategy for the Multinational Large-Scale Piloting of an eHealth Platform: Tutorial on How to Identify Relevant Settings and Use Cases. J. Med. Internet Res. 2023, 25, e42187. https://doi.org/10.2196/42187.
90. De Marco, M.; Cipriani, L.; Baccini, M.; Relli, G.; Livoti, A.; Del Vicario, M.L.; Chiari, L.; Cherubini, G.; Doronzio, S.; Bardi, D.; et al. Test-Retest Reliability of a New Qualitative Pain Assessment Model: Preliminary Results of the WORDSforPAIN Project. Neurorehabilit. Neural Repair 2025, 39, NP117.
91. Di Nicola, M.; Pepe, M.; d'Andrea, G.; Marcelli, I.; Pettorruso, M.; Andriola, I.; Barlati, S.; Carminati, M.; Cattaneo, C.I.; Clerici, M.; et al. Patient Experience with Intranasal Esketamine in Treatment-Resistant Depression: Insights from a Multicentric Italian Study (REAL-ESKperience). J. Pers. Med. 2025, 15, 161. https://doi.org/10.3390/jpm15040161.
92. Doglietto, F.; Vezzoli, M.; Biroli, A.; Saraceno, G.; Zanin, L.; Pertichetti, M.; Calza, S.; Agosti, E.; Aliaga Arias, J.M.; Assietti, R.; et al. Anxiety in Neurosurgical Patients Undergoing Nonurgent Surgery during the COVID-19 Pandemic. Neurosurg. Focus 2020, 49, E19. https://doi.org/10.3171/2020.9.FOCUS20681.
93. Dondi, L.; Ronconi, G.; Dondi, L.; Dell'Anno, I.; Calabria, S.; Addesi, A.; Esposito, I.; Maggioni, A.P.; Martini, N.; Piccinni, C. Il ruolo della Real World Evidence nella fase pre-marketing dei farmaci: Le esperienze e le prospettive future di Fondazione ReS nel delineare i confini delle Target Population. Recenti Prog. Med. 2024, 115, 620–621. https://doi.org/10.1701/4392.43928.
94. Fanelli, G.N.; Ugolini, F.; Ginori, A.; Buccoliero, A.M.; Calcinai, A.; Carnevali, A.; Cassisa, A.; Cappelli, B.; Cattaneo, L.; Cerratani, D.; et al. Toward a Regional Digital Pathology Network in Tuscany: Current Status and Implementation Roadmap. Virchows Arch. 2026. https://doi.org/10.1007/s00428-026-04408-2.
95. Fietta, V.; Rizzi, S.; Gios, L.; Selmi, S.; De Luca, C.; Pederiva, L.; Poggianella, S.; Pavesi, M.C.; Campregher, M.; Lazzeri, S.; et al. Chatbot-Based Version of a World Health Organization-Validated Intervention for Stress Management in Patients With Breast Cancer (Self-Help Plus): Protocol for a Pilot Feasibility Study. JMIR Res. Protoc. 2025, 14, e65837. https://doi.org/10.2196/65837.
96. Garagiola, E.; Bellavia, D.; Schettini, F.; Ferrario, L.; Bonfanti, M.; Porazzi, E.; Rossetto, F.; Blasi, V.; Baglio, F.; Foglia, E. Acceptability of Digital Technologies: The Intention to Adopt T-Cube Solution in COVID-19 Pandemic. Port. J. Public Health 2021, 39 suppl 1, 28. https://doi.org/ 10.1159/000520543.
97. Gastaldi, S.; Tartari, E.; Satta, G.; Allegranzi, B. Advancing Infection Prevention and Control through Artificial Intelligence: A Scoping Review of Applications, Barriers, and a Decision-Support Checklist. Antimicrob. Steward. Healthc. Epidemiol. 2025, 5, e317. https://doi.org/10.1017/ash.2025.10191.
98. Giordani, C.; Di Pumpo, M.; Senarigo, F.; Zambon, C.; Valli, M.; Sbrogiò, L.G.; Milano, N.; Gusella, G.; Zampieri, T.; Allegro, F.; et al. Building AI-Ready Health Systems: The AULSS6 (Padua, Italy) Local Health Authority Artificial Intelligence Implementation Strategy. Ann. Ist. Super. Sanita 2026, 62, 87–90. https://doi.org/10.4415/ANN_26_01_11.
99. Giubilato, S.; Scicchitano, P.; Bilato, C.; Corda, M.; De Luca, L.; Di Marco, M.; Geraci, G.; Iacovoni, A.; Milli, M.; Navazio, A.; et al. Translated Title Show Original ANMCO Position Paper: ANMCO States General 2024 - Role of Cardiologists in the Management of Chronic Cardiovascular Diseases. G. Ital. Di Cardiol. 2025, 26, 861–873.
100. Godoy Junior, C.; Miele, F.; Mäkitie, L.; Bakker, L.J.; Fiorenzato, E.; Uyl-De Groot, C.; Redekop, K.; van Deen, W. PCR103 Patients and Neurologists' Preferences for Remote Patient Monitoring and Artificial Intelligence to Improve Parkinson's Disease Management. Value Health 2022, 25, S410.

101. Iannone, A.; Giansanti, D. Breaking Barriers-The Intersection of AI and Assistive Technology in Autism Care: A Narrative Review. J. Pers. Med. 2023, 14, 41. https://doi.org/10.3390/jpm14010041.
102. Ihaddouchen, I.; Buijsman, S.; Jung, C.; Gommers, D.; Van Genderen, M. Understanding AI Acceptance in European Critical Care: A Cross-Sectional Survey Study Exploring Physician Perspectives. Intensive Care Med. Exp. 2025, 13, 858.
103. Iosca, N.; Monaco, I.P.; Chieppa, A.; Corvascio, M.D.; Lastrucci, A.; Wandael, Y.; Ricci, R. Artificial Intelligence in Radiotherapy: RTTs' Attitudes and Competencies in Two Italian Regions. Radiother. Oncol. 2025, 206, S4252–S4254.
104. Kahlawi, A.; Masri, F.; Ahmed, W.; Vidal-Alaball, J. Cross-Cultural Sense-Making of Global Health Crises: A Text Mining Study of Public Opinions on Social Media Related to the COVID-19 Pandemic in Developed and Developing Economies. J. Med. Internet Res. 2025, 27, e58656. https://doi.org/10.2196/58656.
105. Krakowski, K.; Oliver, D.; Arribas, M.; Logeswaran, Y.; de Micheli, A.; Patel, R.; Stahl, D.; Fusar-Poli, P. Development and Validation of a Precision Treatment Rules for First-Line Antipsychotic Recommendations in First Episode Psychosis Jointly Incorporating Effectiveness, Side Effects and Patient Preferences. Transl. Psychiatry 2026, 16, 252. https://doi.org/10.1038/s41398-026-03914-w.
106. Laborie, L.B.; Naidoo, J.; Pace, E.; Ciet, P.; Eade, C.; Wagner, M.W.; Huisman, T.A.G.M.; Shelmerdine, S.C. European Society of Paediatric Radiology Artificial Intelligence Taskforce: A New Taskforce for the Digital Age. Pediatr. Radiol. 2023, 53, 576–580. https://doi.org/10.1007/s00247-022-05426-3.
107. Lusi, S.; Giannitto, C.; Mercante, G.; Scorsetti, M.; Santoro, A.; Spriano, G.; Balzarini, L.; Politi, L.S. Use of Artificial Intelligence in Management of Head-Neck Tumours: A Multidisciplinary Survey. Insights Into Imaging 2022, 14, 317–318.
108. Magni, P.; Cecchini, I.; Fortunati, M.; Biroli, M.; Massaroni, K.; Folco, E.; Giudice, D.; Montebelli, M.; Ciancamerla, G. Web Discussions on Cardiovascular Diseases: Assessing the Impact of COVID-19 Pandemic. An Italian Prospective Analysis. Eur. J. Prev. Cardiol. 2021, 28, zwab061-441.
109. Maina, S. Artificial intelligence in medical writing: A survey of Italian clinicians' perceptions. Curr. Med. Res. Opin. 2025, 41, S8.
110. Maines, M.; Moz, M.; Forti, S.; Gabardi, E.; Dario, C.; Conforti, D.; Tomasi, G.; Bonmassari, R.; Marini, M.; Del Greco, M. Sustainable Tele-Cardiology System with the Use of Digital - The Trec Cardio Study in Trentino. Eur. Heart J. 2020, 22, G46.
111. Maran, I.; Assenza, M.; Peghetti, A.; Tabarrini, F.; Angelini, G.; Spoltore, G.; Gilli, E.; Casarotti, F.; Chierico, L.; Morganti, A.G.; et al. Current Trends in Therapeutic Radiographers' Research: A Scoping Review. J. Med. Imaging Radiat. Sci. 2025, 56, 101916.
112. Martin, S.; Angolini, E.; Audi, J.; Bertini, E.; Bruno, L.P.; Coulter, J.; Ferlini, A.; Fortunato, F.; Frankova, V.; Garnier, N.; et al. Patient Preferences in Genetic Newborn Screening for Rare Diseases: Study Protocol. BMJ Open 2024, 14, e081835. https://doi.org/10.1136/bmjopen-2023-081835.
113. Mizzi, D.; Allely, C.S.; Zarb, F.; Mercer, C.E. Implementing Supplementary Breast Cancer Screening in Women with Dense Breasts: Insights from European Radiographers and Radiologists. Radiography 2024, 30, 908–919. https://doi.org/10.1016/j.radi.2024.04.003.
114. Monaco, A.; Sforza, G.; Amoroso, N.; Antonacci, M.; Bellotti, R.; de Tommaso, M.; Di Bitonto, P.; Di Sciascio, E.; Diacono, D.; Gentile, E.; et al. The PERSON Project: A Serious Brain-Computer Interface Game for Treatment in Cognitive Impairment. Health Technol. 2019, 9, 123–133.
115. Monaco, F.; Vignapiano, A.; Piacente, M.; Pagano, C.; Mancuso, C.; Steardo, L.; Marenna, A.; Farina, F.; Petrillo, G.; Leo, S.; et al. An Advanced Artificial Intelligence Platform for a Personalised Treatment of Eating Disorders. Front. Psychiatry 2024, 15, 1414439.
116. Montagna, M.T.; De Giglio, O.; Napoli, C.; Fasano, F.; Diella, G.; Donnoli, R.; Caggiano, G.; Tafuri, S.; Lopalco, P.L.; Agodi, A. Adherence to Vaccination Policy among Public Health Professionals: Results of a National Survey in Italy. Vaccines 2020, 8, 379.
117. Nabata, K.J.; AlShehri, Y.; Mashat, A.; Wiseman, S.M. Evaluating Human Ability to Distinguish between ChatGPT-Generated and Original Scientific Abstracts. Updates Surg. 2025, 77, 615–621.
118. Osnat, B. Patient Perspectives on Artificial Intelligence in Healthcare: A Global Scoping Review of Benefits, Ethical Concerns, and Implementation Strategies. Int. J. Med. Inform. 2025, 203, 106007. https://doi.org/10.1016/j.ijmedinf.2025.106007.

119. Oznur, K. EP109 Research Trends and Highlights toward Artificial Intelligence in Pain: Bibliometric Analysis on Web of Science from 2014 to 2023. Reg. Anesth. Pain Med. 2024, 49, A140–A141.
120. Park, J.; Huh, K.; Chung, W.; Lee, S.; Kim, M.; Oh, J.; Yu, K. A Clinical Study to Evaluate the Feasibility of Decentralized Clinical Trial Elements in Korean Patients. Clin. Pharmacol. Drug Dev. 2023, 12, 97.
121. Piazza, D.; Gebbia, V.; Borsellino, N.; Gesolfo, C.S.; Serretta, V. Online Forums and Prostate Cancer Patients: A Descriptive Analysis of Interactions. Anticancer. Res. 2021, 41, 5298–5299.
122. Ramacciati, N.; Metlichin, E.; Giusti, G.D. Exploring the Terminological Validity of "chronic Pain" Nursing Diagnosis: A Retrospective Descriptive Study Using Nursing Diaries. J. Clin. Nurs. 2023, 32, 8032–8042. https://doi.org/10.1111/jocn.16871.
123. Ravaldi, C.; Mosconi, L.; Bonaiuti, R.; Vannacci, A. The Emotional Landscape of Pregnancy and Postpartum during the COVID-19 Pandemic in Italy: A Mixed-Method Analysis Using Artificial Intelligence. J. Clin. Med. 2023, 12, 6140. https://doi.org/10.3390/jcm12196140.
124. Runge, V.M. Current Research and Development in the Field of Magnetic Resonance Contrast Media. Invest. Radiol. 2025, 60, 709–710. https://doi.org/10.1097/RLI.0000000000001206.
125. Seregni, A.; Tropea, P.; Re, R.; Biscaro, V.; Caprino, M.; Judica, E.; Corbo, M. Virtual Coaching System for Continuity of Care and Rehabilitation in Patients with Stroke. Results of the Pilot Study in the Home Scenario. Gait Posture 2022, 97, 29–30.
126. Shahrabani, E.; Nenadic, I.; Aristizabal, S.; Collier, K.M. PRACTICAL APPLICATION OF ARTIFICIAL INTELLIGENCE TO GLOBAL MEDICAL EDUCATION. J. Gen. Intern. Med. 2024, 39, S591.
127. Shalom, E.; Goldstein, A.; Ariel, E.; Sheinberger, M.; Jones, V.; Van Schooten, B.; Shahar, Y. Distributed Application of Guideline-Based Decision Support through Mobile Devices: Implementation and Evaluation. Artifcial Intell. Med. 2022, 129, 102324.
128. Ting, E.; Badin, M.; Gaind, N.; Hofer, K.; Pourrahmat, M.-M.; Ivkovic, L.; Haugli-Stephens, T.; Jacob, J.; Fazeli, M.S. MSR109 Artificial Intelligence Integration in Health Technology Assessments: A Review of Global Policies and Practices. Value Health 2025, 28, S295–S296.
129. Van Cauwenberge, D.; Van Biesen, W.; Decruyenaere, J.; Leune, T.; Sterckx, S. "Many Roads Lead to Rome and the Artificial Intelligence Only Shows Me One Road": An Interview Study on Physician Attitudes Regarding the Implementation of Computerised Clinical Decision Support Systems. BMC Med. Ethics 2022, 23, 50. https://doi.org/10.1186/s12910-022-00787-8.
130. Varrassi, G.; Paladini, A.; Tran, Y.V.; Pham, V.P.; Al Alwany, A.A.; Farì, G.; Caruso, A.; Mercieri, M.; Pergolizzi, J.V.; Kaye, A.D.; et al. Advances in the Pathophysiology and Management of Cancer Pain: A Scoping Review. Cancers 2026, 18, 259. https://doi.org/10.3390/cancers18020259.
131. Vignapiano, A.; Monaco, F.; Landi, S.; Steardo, L.; Mancuso, C.; Pagano, C.; Petrillo, G.; Marenna, A.; Piacente, M.; Leo, S.; et al. Proximity-Based Solutions for Optimizing Autism Spectrum Disorder Treatment: Integrating Clinical and Process Data for Personalized Care. Front. Psychiatry 2024, 15, 1512818.
132. Visci, P.; Sirago, G.; Vinci, A.; Calò, F.; De Micco, F.; Benevento, M.; Solarino, B.; Dell'Erba, A.; Ferorelli, D. Navigating the Landscape of Legal Medicine: A 4-Year Analysis of Forensic Consultations in an Italian Hospital. Front. Med. 2025, 12, 1521195. https://doi.org/10.3389/fmed.2025.1521195.
133. Zavattaro, F.; Barth, C.-M.; Brall, C.; von Wyl, V.; Gille, F. Trust Analysis Canvas for Teaching in the Field of Digital Public Health and Medicine: Tutorial. JMIR Med. Educ. 2026, 12, e79709. https://doi.org/10.2196/79709.
